# Mechanisms of mitophagy and oxidative stress in cerebral ischemia–reperfusion, vascular dementia, and Alzheimer’s disease

**DOI:** 10.3389/fnmol.2024.1394932

**Published:** 2024-08-07

**Authors:** Yujie Lyu, Zhipeng Meng, Yunyun Hu, Bing Jiang, Jiao Yang, Yiqin Chen, Jun Zhou, Mingcheng Li, Huping Wang

**Affiliations:** ^1^Gansu University of Chinese Medicine, Lanzhou, China; ^2^Key Laboratory of Traditional Chinese Herbs and Prescription Innovation and Transformation of Gansu Province, Lanzhou, China; ^3^Laboratory for TCM New Products Development Engineering of Gansu Province, Lanzhou, China; ^4^Xichang Hospital of Traditional Chinese Medicine, Xichang, China; ^5^Qujing 69 Hospital, China RongTong Medical Healthcare Group Co. Ltd, Qujing, China

**Keywords:** mitophagy, oxidative stress, cerebral ischemia/reperfusion, vascular dementia, Alzheimer’s disease

## Abstract

Neurological diseases have consistently represented a significant challenge in both clinical treatment and scientific research. As research has progressed, the significance of mitochondria in the pathogenesis and progression of neurological diseases has become increasingly prominent. Mitochondria serve not only as a source of energy, but also as regulators of cellular growth and death. Both oxidative stress and mitophagy are intimately associated with mitochondria, and there is mounting evidence that mitophagy and oxidative stress exert a pivotal regulatory influence on the pathogenesis of neurological diseases. In recent years, there has been a notable rise in the prevalence of cerebral ischemia/reperfusion injury (CI/RI), vascular dementia (VaD), and Alzheimer’s disease (AD), which collectively represent a significant public health concern. Reduced levels of mitophagy have been observed in CI/RI, VaD and AD. The improvement of associated pathology has been demonstrated through the increase of mitophagy levels. CI/RI results in cerebral tissue ischemia and hypoxia, which causes oxidative stress, disruption of the blood–brain barrier (BBB) and damage to the cerebral vasculature. The BBB disruption and cerebral vascular injury may induce or exacerbate VaD to some extent. In addition, inadequate cerebral perfusion due to vascular injury or altered function may exacerbate the accumulation of amyloid β (Aβ) thereby contributing to or exacerbating AD pathology. Intravenous tissue plasminogen activator (tPA; alteplase) and endovascular thrombectomy are effective treatments for stroke. However, there is a narrow window of opportunity for the administration of tPA and thrombectomy, which results in a markedly elevated incidence of disability among patients with CI/RI. It is regrettable that there are currently no there are still no specific drugs for VaD and AD. Despite the availability of the U.S. Food and Drug Administration (FDA)-approved clinical first-line drugs for AD, including memantine, donepezil hydrochloride, and galantamine, these agents do not fundamentally block the pathological process of AD. In this paper, we undertake a review of the mechanisms of mitophagy and oxidative stress in neurological disorders, a summary of the clinical trials conducted in recent years, and a proposal for a new strategy for targeted treatment of neurological disorders based on both mitophagy and oxidative stress.

## 1 Introduction

Autophagy represents a mechanism by which eukaryotic organisms maintain homeostasis within their intracellular environment. This process involves the degradation of misfolded proteins, necrotic organelles, foreign pathogens and other cellular components. The general process of autophagy involves the separation of damaged organelles from healthy organelles to form autophagosomes, which subsequently fuse with lysosomes to form autophagic lysosomes. These autophagic lysosomes degrade proteins through the action of lysosomal enzymes (Kaushik et al., 2021). In normal circumstances, the level of autophagy within the body is relatively low. However, in response to external stimuli such as starvation, hypoxia and other forms of stress, the number of autophagosomes increases significantly, thereby elevating the overall level of autophagy within the body. Autophagy is classified into three main categories: macroautophagy, microautophagy and chaperone-mediated autophagy. Of these, macroautophagy is the most prevalent form of autophagy. Consequently, macroautophagy is also defined as autophagy in a narrow sense. Macroautophagy is primarily facilitated by double-membrane organelles, including mitochondria, the endoplasmic reticulum, the Golgi apparatus, and the plasma membrane. Mitochondria are of significant importance in the context of energy metabolism within eukaryotic cells. In addition to this, they play a regulatory role in processes such as cell development, the cell cycle and cell death.

The health of mitochondria is of critical importance for neuronal cell function. In recent years, mitophagy has emerged as a mechanism that plays a crucial role in maintaining mitochondrial quality. In order to maintain mitochondrial numbers and physiological functions, the body initiates appropriate mitochondrial biogenesis, damage repair, or autophagic clearance mechanisms under stress, called mitochondrial quality control (MQC) (Song M. et al., 2022). Mitophagy is currently acknowledged as the primary mechanism of MQC in neurons. In the absence of any damage, mitochondria are able to fuse with neighboring intact mitochondria, thereby restoring their functionality (Chan, 2006). Nevertheless, when the extent of mitochondrial damage surpasses the capacity for repair, it results in an alteration in the membrane potential of the outer mitochondrial membrane (OMM), thereby initiating mitophagy. Mitophagy can be broadly categorized into three main types, depending on the physiological condition of the cell: steady-state mitophagy, programmed mitophagy and stress-induced mitophagy (Ke, 2020; Montava-Garriga and Ganley, 2020). The three distinct types of mitophagy serve disparate functions within the cell. Homeostatic mitophagy, which typically occurs under physiological conditions, is responsible for the removal of damaged or aged mitochondria, thereby ensuring the maintenance of mitochondrial quantity and quality. Programmed mitophagy usually refers to the selective degradation of specific mitochondria, a process mostly associated with cell differentiation, development, or other specific physiological processes. Stress-induced mitophagy usually occurs when cells are exposed to external stressors, such as oxidative stress, energy deprivation, or DNA damage. This process prevents cellular damage and apoptosis by facilitating the removal of damaged mitochondria, thereby enabling cells to adapt to stressful conditions. Mitophagy is a process by which damaged or malfunctioning mitochondria are removed from cells. This process is essential for maintaining mitochondrial structural integrity, energy metabolism, regulating the cell cycle and proliferation, preventing oxidative stress, and preventing cell death (Hu et al., 2020). We focus on the phenomenon of stress-induced mitosis. In the event of mitochondrial damage, a reduction in membrane potential is observed, which is followed by the activation of mitophagy genes or factors. This is accompanied by the formation of autophagic vesicles, which wrap around damaged mitochondria to form autophagosomes. These subsequently bind to lysosomes, forming autophagolysosomes, whereby the mitochondria are broken down into amino acids and lipids. Mitophagy is a highly sophisticated process that involves multiple protein complexes and signaling pathways. A detailed description of these will be provided subsequently.

Oxidative stress can be defined as the disruption of the equilibrium between ROS and antioxidant systems within the body. This leads to an accumulation of ROS, which in turn causes damage to proteins, cell membranes, and DNA, while also promoting the development of inflammation and accelerating the process of cellular ageing. Common ROS are Superoxide Anion Radical (O^2−^), Hydroxyl Radical (·OH), Hydrogen Peroxide (H_2_O_2_), Nitric Oxide Radical (NO·), Lipid Peroxyl Radical (Lipid Peroxyl Radical, LOO·), Peroxynitrite Radical (Peroxynitrite, ONOO^−^), and others. The presence of one or more unpaired electrons renders these molecules highly unstable, both structurally and in terms of their reactivity with other molecules. ROS can be generated by many pathways, such as the electron transport chain during mitochondrial oxidative phosphorylation, inflammatory reactions, and UV or radiation exposure, with the electron transport chain during oxidative phosphorylation being the main source of ROS (Lee et al., 2012).

The following section will examine the process of ROS production in mitochondrial oxidative phosphorylation. (1) Glycolysis: within the cytoplasm, glucose is metabolized into two pyruvate molecules, resulting in the generation of small quantities of ATP and reduced nicotinamide adenine dinucleotide (NADH). (2) Pyruvate oxidation and the citric acid cycle: Pyruvate produced by glycolysis is transported to the mitochondria to be converted into acetyl coenzyme A. Acetyl coenzyme A combines with oxaloacetate to form citric acid, which then undergoes a series of chemical reactions that ultimately produce oxaloacetate and NADH, reduced flavin adenine dinucleotide (FADH_2_) and a small amount of ATP. (3) Electron Chain Transfer: NADH and FADH2 transfer electrons to the electron transfer chain in the inner mitochondrial membrane, where protons (H^+^) are pumped from the mitochondrial matrix into the mitochondrial membrane gap in the presence of complexes I, II, III, and IV, as well as auxiliary molecules such as coenzyme Q and cytochrome c (Cyt c), which creates a gradient of protons on both sides of the membrane. (4) Chemically permeable coupling: protons are returned to the substrate by ATP synthase, a process that enables ATP synthase to add phosphate groups to adenosine diphosphate (ADP) to produce ATP. 5. Ultimately, O_2_ combines with electrons and protons to form H_2_O. Throughout this process, the mitochondria can produce approximately 30–32 ATP for every glucose molecule broken down. In typical circumstances, electron transfer is highly efficient. However, a minor leakage of electrons occurs during the transfer processes involving complexes I and III. These leaked electrons reduce oxygen to superoxide anion (O^2−^), which is often rapidly converted by superoxide dismutase (SOD) to H_2_O_2_, which is relatively stable and can cross the cell membrane. Under certain conditions, H_2_O_2_ can further react to form more reactive ROS (e.g., ·OH). ROS cause lipid peroxidation of unsaturated fatty acids in cell membranes, ultimately resulting in the formation of malondialdehyde (MDA), which impairs the function and structure of cell membranes (Mukherjee et al., 2019). In addition, ROS can cause DNA damage, protein oxidation, and induce inflammation and apoptosis (Suski et al., 2012; Wang Y. et al., 2019). In addition to ROS production during electron chain transfer, nicotinamide adenine dinucleotide phosphate (NADPH) oxidase, xanthine oxidase, and dysfunctional endothelial nitric oxide synthase (eNOS) can also produce ROS (Vassort and Turan, 2010). In order to prevent the occurrence of oxidative stress, a variety of antioxidants and enzymes are present in the body to prevent oxidative damage, such as superoxide dismutase (SOD), glutathione (GSH), vitamins C and vitamins E, and so on. Among them, GSH is one of the important reducing agents in the brain, which is oxidized by ROS and converted to glutathione disulfide (GSSG), which is subsequently reduced to GSH by glutathione reductase (Couto et al., 2016). Oxidative stress occurs when the redox imbalance in the body produces ROS that exceed the scavenging capacity of antioxidants. The brain accounts for approximately 20% of the total energy consumed by the entire body, thereby necessitating a higher demand for energy and oxygen. Consequently, the mitochondria within the brain are more active than those found in other tissues. This, coupled with the fact that the brain contains large amounts of unsaturated fatty acids and an imperfect oxidizing enzyme system (e.g., superoxide dismutase, glutathione peroxidase), results in the brain being more susceptible to oxidative stress than other tissues and organs.

Mitophagy and oxidative stress are both intimately associated with mitochondria, and the two are mutually interactive. Collectively, they are implicated in the regulation of cellular function and the maintenance of internal cellular homeostasis. When mitochondria are damaged, the mitophagy program is initiated to prevent the damaged mitochondria from causing damage to the organism; damaged mitochondria are unable to maintain ATP and Ca^2+^ metabolism, leading to a lack of energy and the leakage of electrons, which accelerates the generation of ROS, one of the key factors in the activation of autophagy (Albers and Beal, 2000; Finkel and Holbrook, 2000). Mitochondria are the main site of ROS generation and the main target of ROS. Oxidative stress has been demonstrated to result in impairment of mitochondrial function, including a reduction in mitochondrial membrane potential and damage to mitochondrial membrane integrity. A reduction in mitochondrial membrane potential or damage to the mitochondria can induce mitophagy, which is the process by which damaged mitochondria are removed from the cell, thereby reducing ROS production. A reduction in mitophagy levels, resulting from the inability to remove damaged mitochondria in a timely and effective manner, will lead to an exacerbation of ROS production and accumulation. This, in turn, will cause further damage to the cells, potentially leading to cell death. Mitophagy and oxidative stress are two complex biological processes that are intertwined in multiple signaling pathways. Such as PI3K/AKT/mTOR pathway, AMPK pathway, Nrf2/Keap1 pathway, p53, FOXO, etc. The PI3K/AKT/mTOR signaling pathway is a critical regulator of cellular processes, including growth, metabolism and survival. Mammalian target of rapamycin protein (mTOR) is an important nutrient and energy sensor that plays a role in both oxidative stress and mitophagy. mTOR decreased activity promotes mitophagy while enhancing cellular tolerance to oxidative stress. Adenosine 5-monophosphate (AMP)-activated protein kinase (AMPK) is a key monitor of cellular energy status. AMPK is activated in response to decreased ATP levels, promoting ATP production and inhibiting ATP depletion. p53 is a tumor suppressor protein that plays an important role in DNA damage response and apoptosis. p53 is also involved in the regulation of oxidative stress and mitophagy. p53 in its active form is able to induce mitophagy, and prevents the occurrence of oxidative stress by affecting the expression of antioxidant genes. The active form of p53 induces mitophagy and prevents oxidative stress by affecting the expression of antioxidant genes. The FOXO transcription factor family plays an important role in cellular antioxidant defense and lifespan regulation. Stimulated by ROS, FOXO transcription factors are able to activate a variety of antioxidant genes, including those related to mitophagy. These pathways coordinate the processes of oxidative stress and mitophagy through different mechanisms to maintain normal cellular function and the stability of the intracellular environment.

There is growing evidence that mitophagy and oxidative stress have important roles in the development of neurological diseases. Cerebral ischemia/reperfusion injury (CI/RI), vascular dementia (VaD), and Alzheimer’s disease (AD) are the most common neurological disorders in the clinic. In CI/RI, mitophagy and oxidative stress play important roles. The sudden increase in O_2_ levels after ischemia and hypoxia-reperfusion impairs mitochondrial function and generates a large amount of ROS. ROS initiate mitophagy through the PINK1/Parkin signaling pathway, PI3K/AKT/mTOR signaling, and the FUNDC1 protein to clear damaged mitochondria, thus alleviating oxidative stress and maintaining intracellular environmental homeostasis. In VaD, damage to brain tissue caused by various factors, including insufficient cerebral perfusion, small vessel lesions, and neuroinflammation, can result in oxidative stress, which in turn leads to an excessive accumulation of ROS and further damage to mitochondria. Following the onset of oxidative stress, the mitophagy process is initiated, which results in the removal of damaged mitochondria and a reduction in ROS production. The production of ROS in AD can be accelerated by three main factors: the excessive accumulation of Aβ, excessive phosphorylation of tau protein and inflammation. The BBB is an important functional barrier in the brain and plays a crucial role in maintaining homeostasis of the brain’s internal environment. Among them, tight junction proteins are important proteins for maintaining BBB permeability. Altered BBB permeability is closely related to the development of neurological diseases (CI/RI, VaD, AD, etc.). ROS have the potential to directly damage the endothelial cell membrane of the BBB, leading to cellular damage. Additionally, ROS can disrupt the structure and function of tight junction proteins, including ZO-1, occludin, and claudin, which may result in an increased permeability of the BBB. In conclusion, mitophagy and oxidative stress are of great importance in the physiopathological processes of the organism, and may represent a novel avenue for the treatment of neurological disorders. CI/RI, VaD, and AD, as three common neurological disorders, have a serious impact on the quality of life and the safety of human life. In conclusion, mitophagy and oxidative stress affect the homeostasis of the brain’s internal environment, and a clearer understanding of the cell death mechanisms in neurological disorders, leading to the development of more promising therapeutic agents is imperative and imminent.

## 2 Mechanisms of mitophagy and oxidative stress

### 2.1 Mechanisms of mitophagy

**Figure 1:**
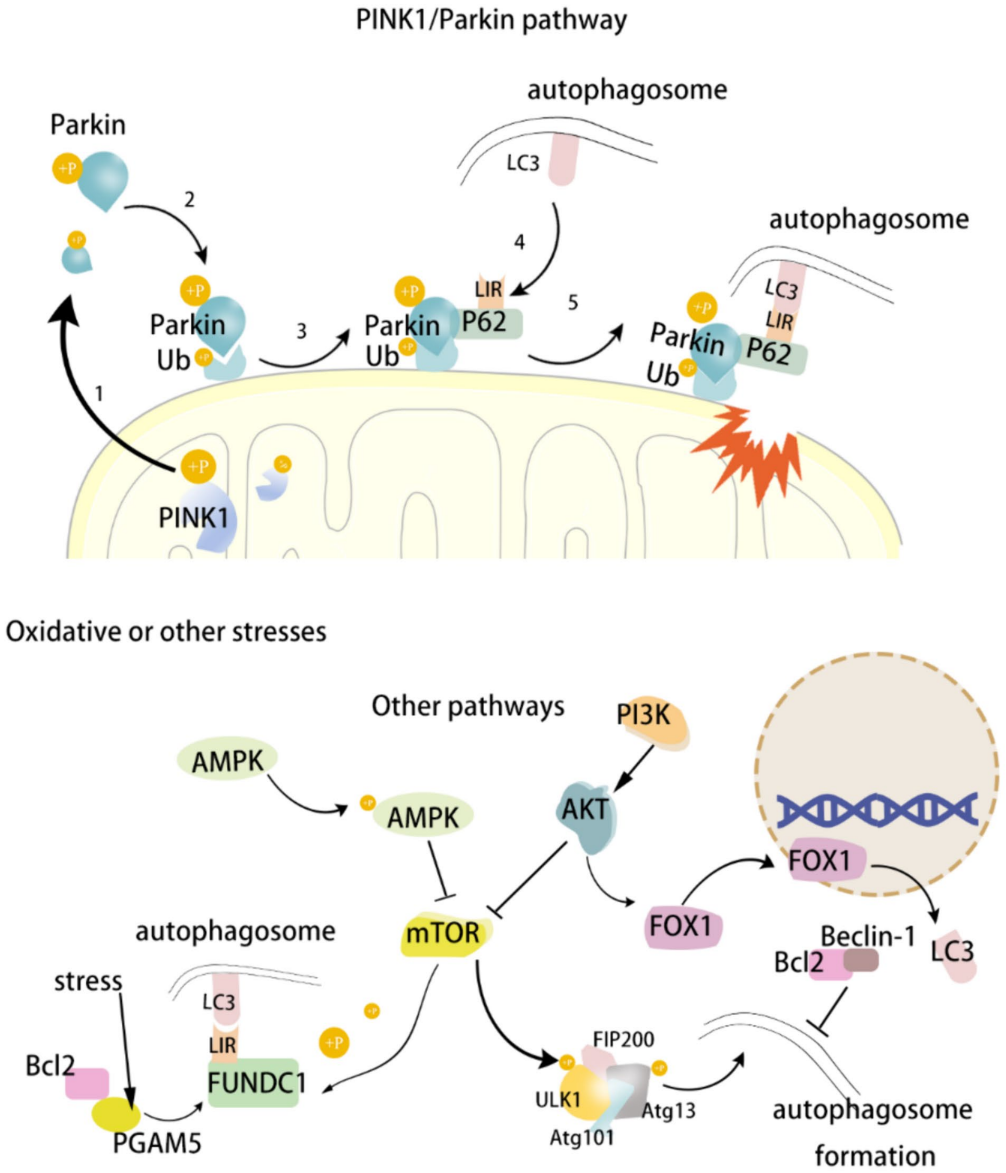
Diagram of mitophagy mechanism.

Mitophagy represents a principal mechanism of MQC, enabling the reduction of cellular damage. A growing corpus of evidence indicates that multiple signaling pathways or factors may be involved in the regulation of mitophagy in the context of the development of neurological diseases. PINK1 has a role in participating in MQC and preventing apoptosis. Parkin is a downstream molecule of PINK1, and the PINK1/Parkin signaling pathway is an important pathway for regulating mitophagy. FUNDC1, an autophagy receptor located in the OMM, regulates mitochondrial fusion/fission and induces mitophagy under hypoxic conditions. Autophagy-related genes (Atg) are important specific genes in the process of cellular autophagy. Mitophagy begins with the activation of the Atg/ULK1 complex, which then activates PI3K, which further activates the PI3K/AKT/mTOR signaling pathway and participates in the regulation of mitophagy. UNC-51-like kinase 1 (ULK1) is an important component of autophagic vesicles and a major regulator of the initiation step of autophagy. The main regulator of autophagy initiation is ULK1. Beclin1 is a key protein in ULK1-activated autophagy. Under normal conditions, the mTOR pathway inhibits Beclin1, thereby reducing mitophagy. On the contrary, under nutrient deficiency or stress conditions, pathways such as AMPK activate Beclin1 and promote mitophagy. There are numerous mechanisms regulating mitochondria, and the above signaling pathways or molecules are only some of them. The aim of this review is to provide a systematic account of the process of regulating mitophagy by the above signaling pathways or molecules, as well as their roles in the occurrence and development of neurological disorders. This will serve to establish a theoretical basis and inspire the development of therapeutic strategies for this class of diseases. (Figure 1).

#### 2.1.1 PINK1/Parkin signaling pathway

The PINK1/Parkin pathway is one of the most important pathways in the mitophagy pathway and most of the mitophagy is mediated by this pathway (Painter et al., 2020). PINK1 is a serine/threonine ubiquitin kinase located primarily in the inner mitochondrial membrane, and its downstream molecule, Parkin, is an E3 ubiquitin ligase (Zhao et al., 2024). Normally, PINK1 is rapidly cleaved and degraded by mitochondrial processing protease (MPP) and proteolytic PINK1 protein [pesenilin-associated rhomboid-like (PARL)], and is therefore extremely low in mitochondria (Geisler et al., 2010; Poole et al., 2010). Under stress, the mitochondrial membrane potential is depolarized and related proteases such as MPP and PARL are inhibited. Originally low levels of PINK1 begin to accumulate and become phosphorylated at the Ser228 and 402 sites, thereby activating PINK1 kinase (Sekine and Youle, 2018). Subsequently, cytoplasmic Parkin translocates to the damaged mitochondrial outer membrane. Mediated by PINK1, Parkin’s Ser 65 site is phosphorylated and initiates its E3 ubiquitin ligase activity, resulting in ubiquitin phosphorylation of mitochondrial outer membrane protein junctions (Harper et al., 2018). Phosphorylated ubiquitin has a high affinity for Parkin, which leads to Parkin recruitment, and newly recruited Parkin will further ubiquitinate mitochondrial outer membrane proteins, thus forming a strong positive feedback loop that amplifies ubiquitin signaling and promotes the aggregation of autophagy receptor 62 (isolation vesicle 1) at the same time (Matsuda and Yamano, 2020). P62 contains an LC3 binding domain (LIR), which helps transport damaged mitochondria to autophagosomes by binding to microtubule-associated protein 1 light chain 3 (LC3) on the autophagosome membrane via LIR. In addition, P62 has a specific ubiquitin-binding domain, UbA (ubiquitin-associated binding domain), a structure that allows P62 to directly recognize and bind to ubiquitinated proteins (Danieli and Martens, 2018). Ubiquitin (Ub) is a small protein that attaches to other proteins by ubiquitination. The process of ubiquitination involves three enzymes: activating enzyme (E1), conjugating enzyme (E2), and ligase (E3), which work in concert to attach ubiquitin molecules to target proteins. During mitophagy, the ubiquitinated protein or organelle is recognized by the p62 autophagy receptor. In conclusion, PINK1 accumulates in mitochondria and phosphorylates at Ser228 and 402 sites, followed by the transfer of Parkin to the OMM and phosphorylates at Ser 65, initiating its E3 ubiquitin ligase activity and phosphorylating the Ub of the mitochondrial outer membrane protein linkage. Then P62 starts to recruit and binds to LC3-II, which sends the damaged mitochondria to autophagosomes and is phagocytosed by autophagosomes. Mature autophagosomes bind to lysosomes to form autophagolytic lysosomes, and eventually the damaged mitochondria are degraded (Lazarou et al., 2015).

#### 2.1.2 FUNDC1 protein

FUNDC1, an autophagy receptor protein located in the OMM and containing a transmembrane LIR structural domain, regulates mitochondrial fusion/fission (DRP1 and OPA1) and induces mitophagy under hypoxic conditions (Chen et al., 2016). In the normal state, FUNDC1 is stably present in the OMM and remains highly phosphorylated (Ser13 and Tyr18 sites phosphorylated) (Greene et al., 2012). FUNDC1 activity is regulated by a variety of signaling pathways. Bcl-2 is primarily involved in apoptosis, but it also plays an important role in the regulation of mitophagy (Dikic and Elazar, 2018). Bcl-2 interacts with phosphoglycerate mutase/protein phosphatase (PGAM5), a member of the phosphoglycerate mutase family, and prevents dephosphorylation of the LIR structural domain of FUNDC1, thereby inhibiting FUNDC1-mediated mitophagy (Wu H. et al., 2014). However, under hypoxia, PGAM5 dephosphorylates the LIR structural domain of FUNDC1 and binds to LC3, thereby inducing mitophagy and helping the cell to remove damaged mitochondria and maintain cellular stability (Wu W. et al., 2014; Chen et al., 2016). LC3 is a key protein in the autophagy process, which is involved in the formation and maturation of autophagosomes. In mitophagy, the interaction between LC3 and FUNDC1 is particularly important, and the LIR domain of FUNDC1 directly binds to LC3 to promote autophagosome formation, encapsulation and disassembly of damaged mitochondria (Wu W. et al., 2014). In addition, AMPK also regulates mitophagy. Activated AMPK under normal conditions inhibits mitophagy by activating AKT, which promotes the phosphorylation of FUNDC1. Under energy-deficient conditions, activated AMPK dephosphorylates FUNDC1, thereby activating mitophagy. In conclusion, FUNDC1, Bcl-2, and LC3 interact with each other to form a complex and sophisticated regulatory system. Dephosphorylated FUNDC1 is able to bind to LC3, thus activating mitophagy. At the same time, Bcl-2 in turn can affect the activity of FUNDC1, thereby regulating the process of mitophagy. This ensures that mitophagy is able to regulate mitochondrial mass in a timely and accurate manner under physiological and pathological conditions, which is crucial for the brain with high energy demands.

#### 2.1.3 PI3K/AKT/mTOR signaling pathway

Autophagy related gene (Atg) is an important gene in the autophagy process, and UNC-51-like kinase1 (ULK1) is the only serine/threonine protein kinase among the 38 known autophagy related proteins, which is an indispensable component of autophagic vesicles. The Atg/ULK1 complex is the major molecule that initiates mitophagy. The Atg/ULK1 complex consists of the interaction of ULK1, family interacting protein of 200 kDa (FIP200), Atg13, and Atg101. The PI3K/AKT/mTOR signaling pathway is an important pathway in the regulation of mitophagy. Phosphoinositide 3-kinase (PI3K) consists of different catalytic subunits, each with a different function. The α-catalytic subunit of PI3K inhibits autophagy while the β-catalytic subunit promotes autophagy (Kma and Baruah, 2022). When ROS are over-accumulated, the β-catalytic subunit of PI3K is activated to produce inositol triphosphate diphosphate (PIP3), and accumulation of PIP3 leads to the recruitment and activation of AKT (also known as protein kinase B) (Wang et al., 2020). Activation of AKT inhibits the downstream mTOR. mTOR is a complex of 2 protein kinases, mTOR1 and mTOR2, that is sensitive to cellular nutrient levels and plays a role in the regulation of cell growth and survival through autophagy. mTOR is a key factor in the inhibition of mitophagy as a key factor in the inhibition of mitophagy. When mTORC1 activity is reduced, it promotes the phosphorylation of ULK1 and Atg13, which negatively regulates the ULK1 complex and promotes the formation of autophagic vesicles. With the activation of the ULK1 complex, Atg9 vesicles can only be recruited to the site of autophagosome formation, thus initiating autophagy. It was shown that cognitive deficits in VaD rats could be improved through the PI3K/Akt/mTOR signaling pathway, which may be related to the increase in the level of mitophagy and the inhibition of neuronal apoptosis, which attenuated neuronal damage and mitochondrial dysfunction (Zheng et al., 2021).

#### 2.1.4 FOXOs gene

FOXOs belong to subgroup O of the forkhead family of proteins, which play important roles in the regulation of cell autophagy, mitochondrial function, proliferation and differentiation, oxidative stress, etc. The FOXOs family contains four members, FOXO1, FOXO3, FOXO4 and FOXO6. Among them, FOXO1 is the only member of the forkhead family of transcription factors with a forkhead structure, and its activity is regulated by PI3K/AKT/mTOR and AMPK signaling pathways (Brunet et al., 1999). The PI3K/AKT/mTOR signaling pathway is associated with cell growth and proliferation. Under physiological conditions, the PI3K/AKT/mTOR signaling pathway is highly active, and activated AKT phosphorylates the Ser25 site of FOXO1, leading to its translocation from the nucleus to the cytoplasm, thus inhibiting its transcriptional activity. Under stress or energy deprivation, AMPK was activated and the above pathway was inhibited. FOXO1 entered the nucleus to activate the expression of LC3 gene and increased the level of mitophagy, thus enabling the cell to maintain energy homeostasis under energy deprivation.

#### 2.1.5 AMPK signaling pathway

AMPK (adenylate-activated protein kinase) regulates mitophagy by directly phosphorylating ULK1 or indirectly acting on the ULK1 complex (Egan et al., 2011). Under hypoxia, lack of energy, or elevated ROS, the intracellular AMP/ATP ratio is elevated, and AMP binds directly to AMPK, thereby activating AMPK (Herzig and Shaw, 2018; Matzinger et al., 2020). AMPK can directly phosphorylate specific amino acid residues (e.g., Ser317 and Ser777) of the ULK1 protein, thereby enhancing ULK1 activity and initiating autophagy. ULK1 forms a complex with several key proteins, such as ATG13, FIP200 (RB1CC1), and ATG101, which together regulate the initiation of autophagy. AMPK can enhance the interaction between ATG13 and ULK1 by phosphorylating ATG13, thereby promoting the stability and function of the autophagy complex (Herzig and Shaw, 2018; Lin and Hardie, 2018). In addition, AMPK activates ULK1 by inhibiting signaling associated with mTORC1. mTORC1 normally inhibits ULK1 under nutrient-sufficient conditions, thereby inhibiting autophagy. When AMPK is activated, it can derepress ULK1 by inhibiting mTORC1 or phosphorylating proteins that interact with mTORC1. At low energy or activated AMPK can promote FUNDC1 dephosphorylation, which activates mitophagy. Studies have shown that activation of AMPK increases FUNDC1 expression (Cai et al., 2021). In contrast, under normal conditions, activated AMPK inhibits mitophagy by activating AKT and promoting phosphorylation of FUNDC1. Metformin is an adenosine monophosphate-activated protein kinase (AMPK) agonist that crosses the BBB and activates AMPK to exert neuroprotective effects (Kersten et al., 2022; Guo et al., 2023). A recent study has shown that metformin is neuroprotective by activating the AMPK/ULK1/PINK1/Parkin signaling pathway and regulating the levels of apoptosis and mitophagy in a hyperglycemic constructed CI/RI animal model and a cellular model of hyperglycemic incubation oxygen–glucose deprivation/reperfusion (OGD/R) (Guo et al., 2023).

#### 2.1.6 Others

Mitochondrial DNA (mtDNA) is a special kind of DNA that exists in the mitochondria of cells. mtDNA is usually a circular double-stranded, unlike the linear DNA in the nucleus. Due to the insufficient repair system of mtDNA and the lack of protective histones, mtDNA is easily damaged when oxidative stress occurs (Kazak et al., 2012). Damaged mtDNA can lead to dysfunction of the mitochondrial respiratory chain, resulting in elevated levels of ROS, which will further damage mtDNA, it was found that ATAD3B contains a LIR motif, which binds to LC3 and promotes mitophagy in a manner that is independent of the PINK1 signaling pathway, resulting in the clearance of the damaged mtDNA (Shu et al., 2021). Under normal conditions, ATAD3B is heterologously coupled to ATAD3A, thereby promoting the targeting of the C-terminal region of ATAD3B to the mitochondrial membrane gap, whereas OS-induced mtDNA damage reduces the heterogeneous heterodimerization of ATAD3B-ATAD3A, leading to the exposure of the C-terminal end of ATAD3B on the mitochondrial outer membrane, which in turn recruits LC3 to initiate mitophagy (Shu et al., 2021).

### 2.2 Mechanisms of oxidative stress

**Figure 2:**
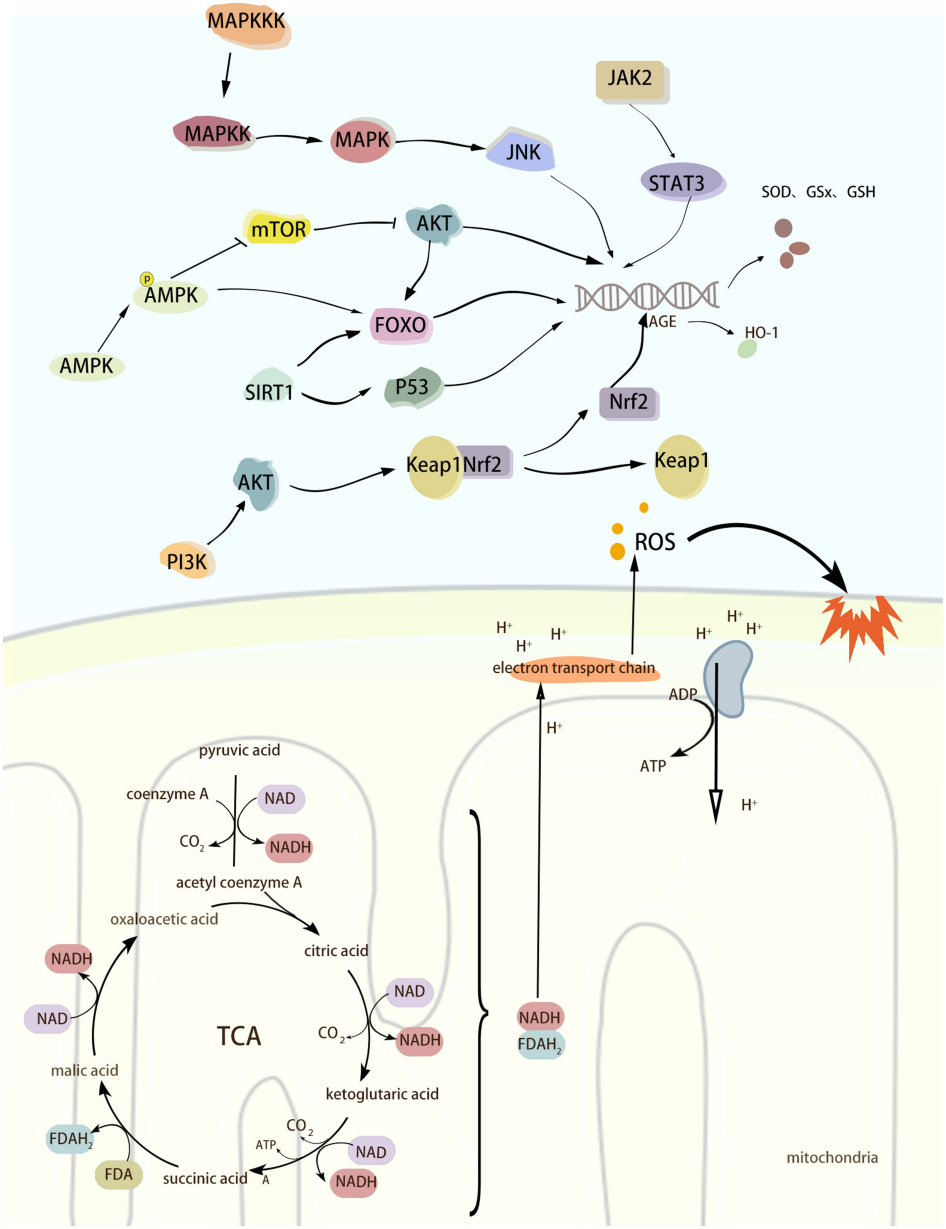
Oxidative stress Schematic diagram of the mechanism.

Increasing evidence suggests that multiple signaling pathways or factors can be involved in the regulation of neurological diseases during the development of oxidative stress. The Nrf2/Keap1 pathway plays an important role in maintaining redox homeostasis and cellular defense mechanisms. Under oxidative stress, Nrf2 dissociates from its repressor Keap1 and activates the expression of antioxidant response genes. ROS can directly activate the JAK2/STAT3 pathway, which can lead to the expression of antioxidant genes, such as superoxide dismutase (SOD) and glutathione peroxidase (GPx), and thus help to scavenge ROS and alleviate the damage caused by oxidative stress. Under hypoxia, lack of energy, or increased ROS, the intracellular AMP/ATP ratio is elevated, and AMP binds directly to AMPK, thereby activating AMPK. activated AMPK phosphorylates Nrf2 and Keap1, which alters their conformation, thereby decreasing the binding capacity of the two, and promotes the displacement of Nrf2 to the nucleus and its binding to the anti-oxidative stress element (ARE) that activates the expression of a series of antioxidant genes. In addition, AMPK can maintain the quality of mitochondria by promoting mitophagy and new mitochondria production, thus reducing the generation of ROS from the root cause. The activation of MAPK/JNK signaling pathway can phosphorylate a variety of transcription factors and promote the expression of antioxidant genes, thus exerting antioxidant effects. In conclusion, there are numerous mechanisms that regulate OS, and the above signaling pathways or molecules are only some of them. The purpose of this review is to present a systematic account of the process of OS regulation by the above signalling pathways and their roles in the development of neurological disorders. The aim is to provide a theoretical basis and inspiration for the development of therapeutic strategies for this class of diseases. (Figure 2).

#### 2.2.1 Nrf2/Keap1 signaling pathway

Nuclear factor erythroid 2-related factor 2 (Nrf2) belongs to the leucine zipper family and includes different functional domains such as Neh1 to Neh7 (Kaspar et al., 2009). Nrf2 is a transcription factor that maintains cellular redox homeostasis and is mainly responsible for regulating the expression of a series of antioxidant response genes to prevent cellular damage caused by ROS. When not activated by ROS, Nrf2 mainly exists in the cytoplasm and binds to Kelch-like ECH-associated protein 1 (Keap1) to form a relatively stable complex, and Keap1 restricts the activity of Nrf2 by promoting ubiquitination and subsequent proteasomal degradation of Nrf2 (Sykiotis and Bohmann, 2008). In the case of oxidative stress occurring with excessive accumulation of ROS, this leads to a decrease in the binding capacity of Nrf2, which is therefore no longer ubiquitinated and degraded but accumulates in the cytoplasm (Li et al., 2018). Keap1 dissociates from Nrf2, followed by Nrf2 nuclear translocation into the nucleus and binding to the antioxidant response element (ARE), which activates the expression of a series of antioxidant and cytoprotective genes, such as Heme oxygenase-1 (HO-1), GST, and GSH, thereby protecting the organism from ROS (He et al., 2020; Chen, 2021). HO-1 has anti-oxidative stress and maintenance of homeostasis in the intracellular environment, and its expression is increased under conditions of oxidative stress, inflammation, heavy metal exposure, and hyperthermia. HO-1 catalyzes the breakdown of hemoglobin into CO, Fe^2+^, and bilirubin. This process removes both heme, which is harmful to the body, and produces bilirubin, which has antioxidant properties, and CO, which is anti-inflammatory and vasodilatory, thus helping to reduce oxidative stress and inflammatory responses and preventing cellular damage. When the level of oxidative stress is reduced, the newly synthesized Keap1 promotes ubiquitination and degradation of Nrf2, and the activity of Nrf2 is re-inhibited, returning the organism to its normal state.

#### 2.2.2 JAK2/STAT signaling pathway

Janus Kinase 2 (JAK2) is a tyrosine kinase belonging to the Janus family that has only one catalytic structural domain and no Src homology 2 (SH2) structural domain. Therefore, JAK2 can not only phosphorylate to bind to other cytokine receptors, but also phosphorylate multiple signaling molecules containing SH2 structural domains (Zhong et al., 2021). Signal Transducer and Activator of Transcription3 (STAT3) is a downstream molecule of JAK2 and belongs to the STAT family of transcription factors. STAT3 consists of an N-terminal structural domain, coiled-coil domain, DNA-binding domain, linker domain, SH2 (Src homology region 2) domain and C-terminal activation domain. Domain, DNA binding domain, linker domain, SH2 (Src homology region 2) domain and C-terminal activation domain. When cytokines (e.g., erythropoietin, interferon, interleukin, etc.) bind to its receptor, they cause a conformational change in the receptor, leading to activation of JAK2 phosphorylation. Activated JAK2 promotes the phosphorylation of STAT3, which undergoes a conformational change to form a dimer and translocate to the nucleus, thereby activating the transcription of antioxidant genes. ROS can directly activate the JAK2/STAT3 pathway, which leads to the expression of antioxidant genes, such as superoxide dismutase (SOD) and glutathione peroxidase (GPx), to help scavenge ROS and reduce the damage caused by oxidative stress.

#### 2.2.3 AMPK signaling pathway

AMPK is a serine/threonine protein kinase complex consisting of an alpha catalytic subunit and two regulatory subunits, beta and gamma (Chen et al., 2018). Under hypoxia, lack of energy, or elevated ROS, the intracellular AMP/ATP ratio is elevated, and AMP binds directly to AMPK, thereby activating AMPK (Herzig and Shaw, 2018). Activated AMPK phosphorylates Nrf2 and Keap1, changing their conformation, thereby reducing their binding capacity, promoting the displacement of Nrf2 to the nucleus and binding to the anti-oxidative stress element (ARE), and activating the expression of a series of antioxidant genes. In addition, AMPK maintains mitochondrial quality by promoting mitophagy and neo-mitochondrial production, which reduces ROS production at the source (Wang et al., 2022). Curcumin promotes Parkin-mediated mitophagy and ameliorates mitochondrial damage through the AMPK/TFEB signaling pathway (Cao et al., 2020). Activation of the AMPK/Nrf2/HO-1 pathway reduces ROS and MDA levels as well as increases SOD and GPx activity and ameliorates CI/RI-induced oxidative stress (Huo et al., 2023). In addition to its antioxidant role by interfering with the Nrf2/Keap1 signaling pathway, AMPK can also play a role by activating FOXO and SIRT1. Nicotinamide adenine dinucleotide phosphate (NADPH) oxidase is another one of the sources of ROS, which produces O^2−^ by catalyzing the oxidation reaction of NADPH. Activated AMPK activates FOXO and SIRT1, which in turn promotes the expression of SOD and glutathione peroxidase, inhibits the activity of NADPH oxidase, and improves cellular antioxidant levels (Tang et al., 2014; Duan et al., 2017; Tang et al., 2021).

FOXO (Forkhead Box O) is a transcription factor that promotes the expression of antioxidant and longevity genes, of which FOXO1, FOXO3, FOXO4, and FOXO6 are involved in the expression of antioxidant genes. AMPK is activated under low energy and hypoxia. Activated AMPK can activate FOXO through phosphorylation, which enhances the transcriptional activity of FOXO and translocates to the nucleus. Promote the expression of antioxidant genes to generate SOD, GPx, glutathione S-transferase (GST), and glutathione synthetase (GCL), which can lighten the cellular damage by oxidative stress (Guo et al., 2017). In addition, AMPK can indirectly regulate the activity of FOXO family proteins through mTOR. As shown previously, mTOR promotes protein synthesis and cell growth mainly through mTORC1 (mTOR complex 1). Under stress, activated AMPK can phosphorylate and activate the activity of TSC2 (Tuberous Sclerosis Complex 2), a component of mTORC1, thus reducing energy consumption. Whereas mTORC1 promotes the activity of AKT kinase, activated AKT phosphorylates FOXO at Ser256, Ser319 and Thr24 sites, which leads to the transfer of FOXO from the nucleus to the cytoplasm, where it binds to phospho-specific proteins and is thus confined to the cytoplasm (Li et al., 2017; Omorou et al., 2023). Since FOXO is required for its initiation of transcription in the nucleus, when FOXO is phosphorylated by AKT it is unable to transcribe antioxidant genes. While AMPK inhibits mTORC1, AKT activity is also inhibited. When AKT activity is low, phosphorylation and extra-nuclear translocation of FOXO protein is reduced, and therefore, FOXO protein can remain in the nucleus to activate the expression of antioxidant genes (Deng et al., 2023).

SIRT1 (Sirtuin 1) and SIRT13 belong to the same sirtuin family, NAD^+^-dependent deacetylases with regulatory and anti-aging effects on oxidative stress (Singh and Ubaid, 2020). AMPK increases intracellular NAD^+^ levels by promoting metabolic pathways such as fatty acid oxidation and glycolysis. NAD^+^ is an essential substrate for SIRT1, and increasing NAD^+^ levels enhances the deacetylation activity of SIRT1. SIRT1 deacetylates a variety of transcription factors, such as FOXO proteins, p53, and NF-κB. FOXO proteins and the p53 transcription factor have a role in the regulation of oxidative stress. After FOXO acetylation, its ability to bind to DNA is reduced, thereby decreasing its transcriptional activity. FOXO has a role in regulating oxidative stress, and the acetylation of FOXO reduces its ability to bind to DNA, thereby decreasing its transcriptional activity. Activated SIRT1 deacetylates FOXO and enhances the transcriptional activity of FOXO, enabling it to bind to DNA more efficiently and activate its downstream target genes of SOD, catalase, and GPx to improve the cellular antioxidant defense capacity (She et al., 2018). In addition, SIRT1 participates in the regulation of oxidative stress by modulating the activity of Tumor Protein 53 (p53) through deacetylation. p53 is a tumor suppressor protein that is widely present in human cells, and its activity can be modulated by acetylation and deacetylation, which enhances the transcriptional activity of p53 and contributes to the activation of apoptosis and cell cycle arrest (Wu et al., 2022). On the other hand, SIRT1 regulates the activity of p53 through deacetylation, thereby modulating the expression of antioxidant-related genes such as glutathione peroxidase 1 (GPX1), SOD2, TP53-induced glycolysis and Apoptosis regulator (TIGAR), and aldehyde dehydrogenase 4 (ALDH4), and reduces the cellular damage caused by ROS (Liu et al., 2020). In summary, AMPK plays an antioxidant role by activating the Nrf2/HO-1 signaling pathway and regulating FOXO and SIRT1 factors to promote the expression of antioxidant genes. AMPK also maintains the quality of mitochondria by promoting mitophagy and neo-mitochondrial production, which reduces ROS production from the root. Mitochondrial deacetylase Sirtuin-3 (SIRT3) has an antioxidant effect. Cellular experiments have demonstrated that Aβ decreases the level of SIRT3 in neurons, whereas a lack of SIRT3 leads to increased levels of oxidative stress, neuronal hyperexcitability, and a decrease in survival, and that the use of SIRT3 agonists reduces the level of ROS and inhibits neuronal hyperexcitability (Ying et al., 2022). Also, animal experiments showed that inhibition of SIRT3 resulted in elevated neuronal excitability, whereas decreasing ROS levels reversed the neuronal hyperactivity caused by the lack of SIRT3, and these findings suggest that SIRT3 regulates the level of oxidative stress in AD neurons (Ying et al., 2022).

#### 2.2.4 MAPK/JNK signaling pathway

MAPK kinase kinase (MAPKKK), MAPK kinase (MAPK kinase, MAPKK) is the upstream molecule of the MAPK signaling pathway. JNK belongs to the MAP kinase (mitogen-activated protein kinase) family of kinases, which plays an important role in a variety of physiological and pathological processes, such as cell cycle, reproduction, apoptosis and cellular stress. It plays an important role in various physiological and pathological processes including cell cycle, reproduction, apoptosis and cellular stress, especially in the process of oxidative stress (Johnson and Lapadat, 2002). There are three isoforms of the JNK family, including JNK1, JNK2, and JNK3. When oxidative stress occurs, ROS can activate JNK by affecting upstream kinases of JNK, such as MAPKKK and MAPKK. ASK1 (Apoptosis Signal-regulating Kinase 1) belongs to an isoform of MAPKKK, also known as MAPKKK5, and can be activated by ROS, tumor necrosis factor-alpha (TNF-alpha), and lipopolysaccharide, among others. Trx is an ASK1-binding protein, and inhibits the activity of ASK1 through binding to it (Matsuzawa and Ichijo, 2008). Under oxidative stress, the sulfhydryl groups in the two cysteine residues contained in Trx are oxidized to form a disulfide bond, which separates Trx from ASK1 and induces the aggregation and activation of ASK1, which in turn activates downstream JNK (Matsuzawa and Ichijo, 2008; Katagiri et al., 2010). JNK activation phosphorylates a variety of transcription factors and promotes the expression of antioxidant genes, thereby exerting an antioxidant effect. Activation of JNK/SAPK in response to oxidative stress mediates the activation of BACE, which in turn leads to elevated Aβ levels (Tamagno et al., 2005, 2006).

## 3 The role of mitophagy and oxidative stress in neurological disorders

### 3.1 The role of mitophagy in neurologic diseases

**Figure 3:**
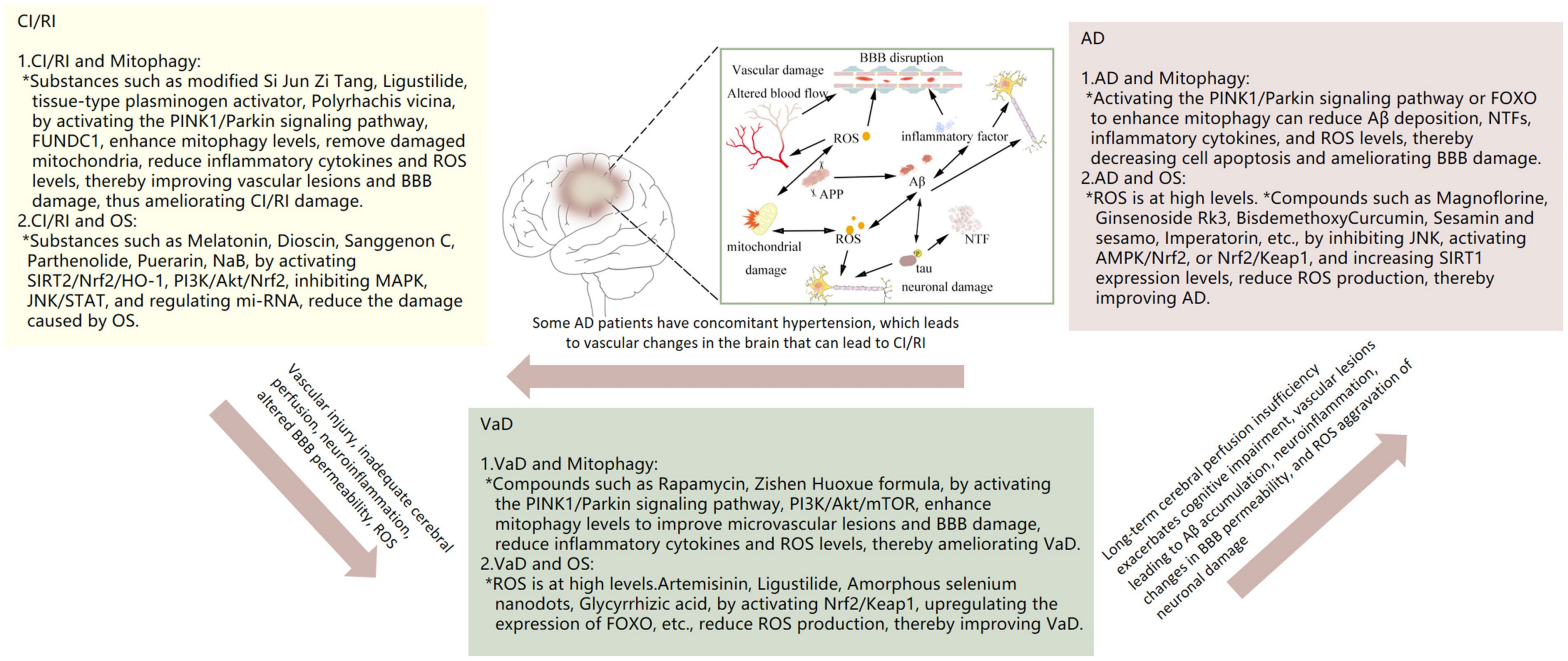
Relationship of mitophagy, OS with CI/RI, VaD and AD.

Mitophagy represents a crucial process in MCQ, whereby the removal of damaged mitochondria is facilitated. This process plays a pivotal role in maintaining intracellular homeostasis and enabling cells to respond effectively to environmental stresses. The relationship between mitophagy and neurological disorders (e.g., CI/RI, VAD, AD) is currently the subject of extensive research. A number of studies have indicated that mitophagy may be a significant factor in the development and progression of neurological disorders. For example, CI/RI has been demonstrated to result in oxidative stress and neuroinflammation, which in turn cause damage to cells. Mitophagy can maintain intracellular environmental homeostasis by removing damaged mitochondria and reducing ROS production. More and more studies have shown that mitophagy plays a complex role in the development and progression of VaD and AD, which may be related to factors such as inflammation, oxidative stress, vascular injury, and abnormal vascular function. However, the relationship between mitophagy and neurological disorders, especially CI/RI, has not been studied deeply enough, and in-depth studies are still needed to reveal the specific mechanisms. We conducted a systematic review of the role of mitophagy in the development of three neurological diseases: CI/RI, VaD, and AD. We then provided strategies for intervening in neurological diseases from the mitophagy pathway (Table 1 and Figure 3).

**Table 1:** Experimental Study on Correlation between CI/RI, VaD and AD.

No.	Diseases	Model	Intervention drug or modality	Machine	Reference
1	CI/RI	Middle cerebral artery focal cerebral ischemia (MCAO) rat model	The Modified Sijunzi decoction	It regulates mitophagy, protects mitochondrial homeostasis, enhances mitochondrial autophagy, improves CI/RI, and reduces mNSS neurological function scores in rats through the PINK1/Parkin signaling pathway.	Song et al. (2023)
2	CI/RI	Middle cerebral artery occlusion and reperfusion (MCAO/R) rat model and oxygen–glucose deprivation reperfusion (OGD/R) cell model	Ligustilide (LIG)	Promoting mitophagy and improving mitochondrial function through PINK1/Parkin, thereby alleviating neuronal damage.	Mao et al. (2022)
3	CI/RI	MCAO rat model	Electroacupuncture, cyclosporine-A (CsA, a potent inhibitor of mPTP opening) or FeTMPyP (a type of ONOO scavenger)	It increases mitophagy by modulating the Pink1/Parkin signaling pathway. Ameliorates CI/RI-induced impairment of mitochondrial function, such as reduced mitochondrial membrane potential (MMP) and ATP levels, and aggregation of damaged mitochondria. Reduces ALP dysfunction mediated by excessive nitroxyl/oxidative stress and PI3K/Akt/mTOR signaling pathway.	Wang H. et al. (2019)
4	CI/RI	MCAO rat model, bEndOGD/R cell model	Active fraction of Polyrhachis vicina (Roger) (AFPR)	It significantly reduced neural scores and infarct area in CI/RI rats, which may be associated with increased microvessel density and VEGFA expression, increased SIRT3 expression, and activation of Pink1/Parkin-mediated mitophagy to significantly promote angiogenesis; attenuated neuronal apoptosis in the cerebral cortex, and increased the density of Nissl in the hippocampus.	Wei et al. (2023)
5	CI/RI	MCAO Pnn-deficient mice model		Pnn deficiency leads to oxidative stress and increased area of cerebral infarction and elevated expression of pro-apoptotic proteins.	Hsu et al. (2022)
6	CI/RI	transient middle cerebral artery occlusion (tMCAO) mice	Melatonin	It promotes SIRT3 expression and activates the SIRT3 signalling pathway to attenuate brain damage.	Liu et al. (2019)
7	CI/RI	MCAO rat model, OGD/R-induced PC12 cell model	Sanggenon C (SC)	It exerts neuroprotective effects by modulating the RhoA/ROCK signaling pathway, inhibiting inflammation and reducing the level of oxidative stress.	Zhao and Xu (2020)
8	CI/RI	OGD/R-induced PC12 cell model	Parthenolide (PN)	It exerts neuroprotective effects by activating the Akt/GSK-3β signalling pathway, inhibiting oxidative stress, significantly reducing HIF-1α expression, and inhibiting apoptosis.	Zhang et al. (2017)
9	CI/RI	MCAO rat model, OGD/R-induced HT22 cell model	Puerarin (PUE)	It activates Nrf2 and promotes the expression of downstream antioxidant enzymes through the PI3K/Akt signalling pathway, thereby inhibiting ROS release, ameliorating oxidative stress and ameliorating neuronal damage.	Zhang et al. (2023)
10	CI/RI	Bilateral common carotid artery occlusion (BCCAO) mice model	sodium butyrate (NaB)	It reduces oxidative stress by inhibiting the JNK/STAT pathway, increasing SOD activity, and inhibiting MDA activity; reduces inflammation by decreasing levels of IL-1β, TNF-α, and IL-8; and inhibits neuronal apoptosis.	Wang R. X. et al. (2019)
11	VaD	2-VO rat model	Tongmai Kaiqiao Pil	It promotes mitophagy by activating the HIF-1α/BNIP3 signalling pathway, thus removing functionally impaired mitochondria, providing energy for healthy cells, reducing neuronal cell death, and promoting the recovery of brain function, thus reducing ischaemic damage in rat hippocampal tissue and improving learning and memory ability, thus exerting a therapeutic effect on VaD。	Ding et al. (n.d.)
12	VaD	Bilateral carotid artery permanent ligation rat model	Rapamycin (RAPA)	It inhibited neuronal apoptosis and enhanced mitophagy through the PI3K/AKT/mTOR pathway. Thereby, it improved cognitive impairment and attenuated neuronal damage and mitochondrial dysfunction in VaD rats.	Zheng et al. (2021)
13	VaD	2-VO rat model	Zishen Huoxue Decoction (ZSHXD)	It ameliorates mitochondrial structural damage and dysfunction in the CA1 region of the hippocampus by activating the PINK1/Parkin signalling pathway, increasing the activity of SOD, decreasing the activity of MDA, inhibiting the apoptosis of Caspase-3, decreasing the level of Bax, and increasing the level of Bcl-2, thereby alleviating the neuronal damage.	Zhao et al. (2024)
14	VaD	VaD patients	Zishen Huoxue Decoction (ZSHXD)	It improved MMSE scores, ADL scores, TCM evidence scores, and NIHSS scores in VaD patients.	Wu et al. (2015)
15	VaD	2-VO at model, hydrogen peroxide stress cells	Puerarin (PUE)	It protects against vascular dementia by reducing MDA levels, inhibiting ROS production, increasing glutathione peroxidase and total thiol levels, and up-regulating Nrf2, FOXO1, FOXO3, and FOXO4, thereby reducing oxidative stress and improving learning and memory functions.	Zhang et al. (2015)
16	VaD	BCCAO rat model	*Artemisia annua* Linné (AA)	It protects neurons by activating the Nrf2/Keap1/ARE signaling pathway, reducing levels of oxidative stress and neuroinflammation, attenuating cortical microvessel and BBB damage, and protecting neurovascular unit (NVU) integrity.	Kim et al. (2023)
17	VaD	BCCAO rat model	Ligustilide (LIG)	It reduces the level of oxidative stress, decreases the level of Bax and increases the level of Bcl-2 in the rat brain by activating the AMPK/SIRT1 signaling pathway, thus improving the learning and memory ability of rats.	Peng et al. (2022)
18	VaD	BCCAO rat model	Amorphous selenium nanodots (A SeNDs)	It improves the learning memory ability of rats by regulating the NMDAR signalling pathway, restoring blood flow in the posterior cerebral artery, improving the neuronal morphology and dendritic remodelling of pyramidal cells in the CA1 region of the hippocampus, decreasing the level of oxidative stress in the rats, increasing the expression of the NR2A, PSD95, and CaMK II proteins, and decreasing the concentration of intracellular calcium ion, and thus improving the learning memory ability of the rats.	Zhu et al. (2023)
19	VaD	2-VO rat model	Glycyrrhizic acid	It inhibits ROS production, reduces cytochrome-c release and decreases apoptosis by modulating the GSK3β/Nrf2 signaling pathway and restoring mitochondrial complexes I and IV, enzymatic and non-enzymatic antioxidant activities.	Sathyamoorthy et al. (2020)
20	AD	AD patient, AD cell model, Cryptobacterium hidradii AD model, APP/PS1 mice model	NAD supplements, urolithin A and actin	It attenuates cognitive impairment in APP/PS1 model mice by increasing the level of mitophagy and phagocytosis in microglia, decreasing the levels of Aβ_1-42_ and Aβ_1-40_, decreasing tau hyperphosphorylation, and inhibiting neuroinflammation via the PINK1/parkin signaling pathway or the DCT-1-dependent pathway.	Fang et al. (2019)
21	AD	mTau-HT22 cell	urolithin A, actinonin, tomatidine, nicotinamide riboside	It increased cell viability and raised mRNA and protein levels of mitochondrial fusion, synaptic and mitophagy genes.	Kshirsagar et al. (2021)
22	AD	Apoe4 mice, BV2 cell and HT22 cell transfected with Apoe4	3,14,19-triacetylandrographolide (ADA)	It improves mitophagy via the SIRT3-FOXO3a signaling pathway, which in turn inhibits NLRP3 inflammatory vesicles to attenuate AD pathology.	Zhou et al. (2024)
23	AD	APP/PS1 mice	UMI-77	MCL-1 improves learning memory in APP/PS1 mice by inducing mitophagy.	Cen et al. (2020)
24	AD	APP/PS1 mice, Aβ-induced PC12	Magnoflorine	It reduces apoptosis, increases SOD levels, and decreases MDA and ROS levels by inhibiting the JNK signalling pathway.	Zhong et al. (2023)
25	AD	APP/PS1 mice	Ginsenoside Rk3 (Rk3)	It inhibits ROS production by activating the AMPK/Nrf2 signalling pathway, restores mitochondrial membrane potential, increases GSH and SOD levels, inhibits MDA production and reduces apoptosis.	She et al. (2023)
26	AD	APP/PS1 mice	Bisdemethoxycurcumin (BDMC)	It inhibits oxidative stress, increases the number of neurons, and reduces Aβ deposition by increasing the level of SIRT1 expression, thereby improving the learning memory ability of APP/PS1 mice.	Xu et al. (2020)
27	AD	Cobalt chloride-induced chemical hypoxia and hypoglycaemia in hippocampal neuronal cells as a model of vascular dementia *in vitro*	Imperatorin	It reduces oxidative stress, decreases mitochondrial membrane potential, and ameliorates cobalt chloride-induced apoptosis by modulating the Nrf2/HO-1 signaling pathway.	Liao et al. (2023)

#### 3.1.1 Mitophagy and CI/RI

CI/RI is a form of brain tissue damage that occurs as a result of the recirculation of blood in the brain following a period of cerebral ischemia and hypoxia (Salameh and Dhein, 2015; Liu et al., 2023). This leads to a range of cognitive impairments, including reduced learning and memory abilities. The Circle of Willis is a ring of arteries located at the base of the brain, consisting of the anterior cerebral arteries, middle cerebral arteries, posterior cerebral arteries, basilar arteries, and communicating arteries, which equalize and distribute blood flow from the internal carotid arteries and vertebral arteries (Mastorakos and McGavern, 2019). The anterior, middle, and posterior cerebral arteries and their branches form abundant cortical arteries and arterioles on the surface of the brain. Cortical artery branches penetrate deep into the brain parenchyma, giving rise to smaller arterioles, and the perivascular space has become an important site for the removal of unwanted proteins and peptides (e.g., Aβ) (Gorelick et al., 2011; Laman and Weller, 2013). The vascular structure of the brain, especially its “outside-in” blood supply, places the deeper part of the brain at the junction of the two main blood supply areas - the watershed areas - which are most susceptible to ischemic injury in the event of a drop in blood pressure or a decrease in blood flow.

There are 8.6 billion neurons and 25–30 billion glial cells in the brain, making the brain the organ that consumes the most oxygen (Huang et al., 2023). Compared with muscle and liver, the brain has relatively insufficient energy reserves, which largely depends on blood supply, and is therefore extremely sensitive to ischemia. When ischemia and hypoxia occur, ATP synthesis in the brain is reduced, neurons are depolarized, excitatory amino acids are released from the presynaptic membrane, excitatory amino acids are reduced from the postsynaptic membrane, and Ca^2+^ is overloaded, which ultimately results in the gradual death of neurons and produces structural damage to the brain tissue. During reperfusion, the above damage is further aggravated, resulting in mitochondrial damage and the production of large amounts of inflammatory factors and ROS (Li et al., 2015). In conclusion, the series of reactions mentioned above (ROS, Ca^2+^ overload and excitatory amino acid damage, etc.) can damage the structure of mitochondria, cause mitochondrial dysfunction and activate intracellular mitophagy. In the acute phase of ischemia, mitochondrial damage was severe but no obvious mitophagy was detected; however, with the prolongation of ischemia over a certain period of time, the degree of mitophagy increased (Orellana-Urzúa et al., 2020). In the early stage of cerebral ischemia–reperfusion injury, damaged mitochondria can be cleared in time by mitophagy, which reduces the production of ROS and thus exerts neuroprotective effects. Under normal conditions, mitophagy helps to maintain the energy metabolism and barrier function of BBB endothelial cells. However, after the occurrence of CI/RI the level of inflammatory factors in the brain increased, and BBB permeability increased (Choi et al., 2014). Microglia are the main immune cells in the nervous system. Microglia are activated in the early stage of CI/RI and play the roles of phagocytosis of damaged and dead cells, release of inflammatory factors and cytokines. Inflammatory factors such as TNF-α, IL-1β, and IL-6 bind to the target cells through specific receptors and activate the downstream inflammatory signaling pathways (e.g., the NF-κB signaling pathway), which further promote inflammatory factors and chemokine expression. Inflammatory factors such as TNF-α, IL-1β and IL-6 bind to target cells through specific receptors and activate downstream inflammatory signaling pathways (e.g., NF-κB signaling pathway), further promoting the expression of inflammatory factors and chemokines, exacerbating inflammatory responses, inducing apoptosis and necrosis, and resulting in brain tissue damage. However, sustained high levels of inflammatory factors induce or exacerbate oxidative stress and mitochondrial dysfunction, inhibit mitophagy, and cause greater tissue damage. In the case of CI/RI, inflammatory factors or ROS cause neuronal death and impairment of the structural integrity of the BBB, leading to easier entry of blood components and inflammatory factors into the brain tissue and exacerbating brain damage (Zhong et al., 2022). In conclusion, in CI/RI, neuroinflammation, mitophagy, and oxidative stress are not isolated from each other; there is a complex interplay between them, and the complex mechanism between them determines the degree of brain tissue damage.

Studies have shown that Jiawei Sijunzi Tang can significantly improve the neurological function, reduce mNSS score, maintain mitochondrial homeostasis, increase the mitochondrial membrane potential level and Ca^2+^-Mg^2+^-ATPase activity, and reduce the O^2−^ content in the CI/RI model rats through the PINK1/Parkin signaling pathway (SONG et al., 2023). When the PINK1/Parkin signaling pathway is activated, it promotes mitochondrial autophagy, inhibits the activation of NLRP3 inflammatory vesicles and reduces the level of inflammation (Fan et al., 2021). Both *in vivo* and *in vitro* experiments demonstrated that Ligustilide (Chuanxiong Lactone) promotes mitophagy via PINK1/Parkin, thereby ameliorating neuronal injury in CI/RI. Therefore, neuronal injury can be ameliorated by targeting intervention in the PINK1/Parkin signaling pathway to promote mitophagy (Mao et al., 2022). Therefore, neuronal injury can be ameliorated by targeting intervention in the PINK1/Parkin signaling pathway to promote mitophagy. Electroacupuncture (EA) is a method of preventing and treating diseases by using a combination of acupuncture and electrical stimulation, which has the effect of increasing synaptic plasticity and improving the level of neurotrophic factors, after the needle is inserted into the acupoint to obtain Qi (Lai et al., 2019). EA was found to enhance Pink1/Parkin-mediated mitochondrial autophagic clearance, reduce accumulation of damaged mitochondria, and ameliorate nitro/oxidative stress-induced functional impairment of brain CI/RI mitochondria (Wang et al., 2019). Tissue-type plasminogen activator (tPA), the only FDA-approved clinical thrombolytic agent, exerts neuroprotection by activating AMPK to increase FUNDC1 expression, thereby inhibiting apoptosis and improving mitochondrial function (Cai et al., 2021). Polyrhachis vicina (Roger) increased the level of mitophagy and promoted angiogenesis through activation of the SIRT3-mediated Pink1/Parkin signaling pathway, significantly reduced neurological scores and infarct area, alleviated cortical neuron apoptosis, and increased hippocampal Nissl density in CI/RI rats (Wei et al., 2023).

#### 3.1.2 Mitophagy and VaD

VaD is a cognitive impairment caused by various cerebrovascular diseases and is one of the most common forms of dementia, accounting for 20% of all cases. As life expectancy increases and the incidence of cardiovascular diseases continues to rise, it is likely that the prevalence of VaD will continue to grow in the coming decades (Wu et al., 2016). The primary clinical manifestations of vascular dementia (VaD) include cognitive impairment and motor abnormalities, which have a significant adverse impact on the quality of life of the patients (Gorelick et al., 2011; O'Brien and Thomas, 2015). There are many causes of VaD, such as insufficient cerebral perfusion, oxidative stress, neuroinflammation, vascular lesions in the brain (atherosclerosis, endothelial dysfunction, and cerebral small-vessel disease, etc.), alterations in the permeability of the blood–brain barrier (BBB), and cholinergic damage (Sharp et al., 2009; Iadecola, 2013; Liu et al., 2017). However, reduced cerebral blood supply due to ischemic or hemorrhagic brain lesions, for example, thus leaving the brain in a state of chronic cerebral hypoperfusion (CCH) is the most common cause of VaD (Wang et al., 2010; Iadecola, 2013). Decreased cerebral blood flow (CBF) can lead to changes in spatial memory (Sigfridsson et al., 2020), CCH can cause some damage to brain tissue, such as lacunar infarcts, brain atrophy, white matter damage, microhemorrhages, and microinfarcts, which can lead to brain dysfunction and cognitive impairment (Alosco et al., 2013). Study shows reduced serum levels of Parkin protein in patients with VaD and AD (Castellazzi et al., 2019). Rapamycin (RAPA) improved cognitive deficits and attenuated neuronal damage and mitochondrial dysfunction in VaD rats by increasing the level of mitophagy and inhibiting neuronal apoptosis via the PI3K/Akt/mTOR signaling pathway (Zheng et al., 2021). ZiShenHuoXue Formula (ZSHXF) is composed of He Shouwu, Chinese wolfberry, mulberry, Schisandra chinensis, Salviae Miltiorrhizae, Puerariae Root, Yizi Ren, *Acorus calamus*, Ulmus, Preparation of Yuanzhi, Scorpion, Hawthorn, and it is an empirical formula for the treatment of VaD, which was formed under the guidance of the master of national medicine Liu Zuyi, and has exact clinical efficacy (Zhou et al., 2004; Wu et al., 2015). It was also shown that ZSHXD could attenuate neuronal damage through PINK1/Parkin-mediated mitophagy and exert neuroprotective effects in VaD rats (Zhao et al., 2024).

#### 3.1.3 Mitophagy and AD

AD is a neurodegenerative disease that mostly occurs in the elderly population, and its main clinical manifestations are memory loss, cognitive impairment, language impairment and accompanied by depression, apathy and other emotions, which make it difficult to take care of oneself in daily life, and bring a heavy burden to patients and their families. Aging is one of the most important factors in the development of AD. With the aging of the world’s population, the incidence of AD is on the rise year by year, and it is expected that by 2050, there will be as many as 152 million AD patients in the world, and the cost of treating AD will be as high as 1.1 trillion U.S. dollars by then (Orobets and Karamyshev, 2023; Yang et al., 2023b). The main pathologic manifestations of AD are amyloid-β (Aβ) formation of senile plaques, tau protein formation of neurofibrillary tangle (NTF), neuronal damage and synaptic dysfunction (Yeo et al., 2014). Numerous studies have shown that neuroinflammation is another important factor exacerbating AD. Microglia (MG) are important immune cells in the central nervous system, which can recognize and remove pathogens, damaged cells to maintain the role of brain microenvironmental homeostasis. In the early stages of AD, MGs have a role in recognizing and clearing Aβ deposits. However, as AD progresses, MGs are in an over-activated state, releasing pro-inflammatory cytokines and exacerbating the pathologic process of AD. Thus, MG plays a complex role in the pathophysiology of AD, both as a defense mechanism in the early stages of the disease and as a facilitator in the late stages of the disease. Higher levels of inflammatory factors inhibit mitophagy, and in AD, low levels of PINK1 and Parkin lead to impaired mitophagy (Pradeepkiran and Reddy, 2020; Tran and Reddy, 2020; Morton et al., 2021). Increased levels of mitophagy were found to reduce Aβ deposition and NTF in human neuronal cells and improve memory deficits in AD model mice (Fang et al., 2019). Synaptophysin, a membrane glycoprotein mainly found in the synaptic vesicle membrane of neurons, has a role in transmitting neural signals and regulating synaptic plasticity. Treatment with mitophagy enhancers resulted in a significant increase in the cell survival rate of mutant tau-HT22 cells, as well as an increase in the mRNA and protein levels of both synaptophysin and mitophagy genes (Kshirsagar et al., 2021). Studies have shown cerebrovascular dysfunction in patients at risk for AD or in early stages of AD, suggesting that inadequate cerebral perfusion is associated with the development of AD (Iadecola, 2004; Claassen et al., 2009; Sabayan et al., 2012; Gao et al., 2013). In the presence of inadequate cerebral perfusion and hypoxia, β-secretase is activated and tau protein is hyperphosphorylated (Kitaguchi et al., 2009; Koike et al., 2010). By increasing the level of mitophagy, Aβ deposition can be reduced and the phagocytosis and anti-inflammatory effects of microglia can be modulated, which in turn attenuates cognitive deficits in APP/PS1 mice; mitophagy also inhibits the overphosphorylation of tau proteins and ameliorates the memory deficits in the genetically modified nematode Hidradenitis elegans cryptic rod nematode and APP/PS1 mice (Fang et al., 2019). An acetylated derivative of Andrographolide (3,14,19-triacetylandrographolide, ADA) ameliorates cognitive deficits and reduces Aβ deposition and neuronal damage in Apoe4 mice by improving mitochondrial autophagy via the SIRT3/FOXO3a signaling pathway, which in turn inhibits NLRP3 inflammatory vesicles (Zhou et al., 2024). Experiments showed that MCL-1 is a mitophagy receptor that directly binds to LC3A, while UMI-77 is a mitophagy activator that can be targeted to induce mitophagy. UMI-77 effectively induced mitophagy at a sublethal dose and improved the cognitive deficits in APP/PS1 mice, reduced the inflammatory response and the pathologic damages induced by Aβ plaques, and promoted the damaged mitochondria clearance of damaged mitochondria (Cen et al., 2020). The amyloid precursor protein intracellular domain (AICD) is a transcription factor that regulates the transcription of multiple genes, and AICD interacts with FOXO3a in the nucleus to regulate the transcription of the PINK1 promoter (Goiran et al., 2018). Furthermore, modulation of γ-secretase activity or AICD expression affects Pink1-mediated mitophagy (Goiran et al., 2018).

### 3.2 The role of oxidative stress in neurological disorders

In recent years, the research on oxidative stress and neurological diseases (including CI/RI, VaD and AD) has been gradually deepened. Several studies have shown that oxidative stress plays an important role in the development and progression of neurological diseases. CI/RI leads to abnormal mitochondrial function and massive ROS production, resulting in oxidative stress and cellular damage. A study on oxidative stress and dementia showed that patients with AD and VaD had higher levels of oxidative stress than normal; MDA levels were more than 2.8 times higher in VaD patients than in AD patients (Gustaw-Rothenberg et al., 2010). Oxidative stress is one of the important pathogenic factors of VaD and AD, ROS can trigger inflammation, cellular damage, vascular damage, abnormal vascular function and thus lead to insufficient blood supply to the brain, thus inducing VaD; in the brains of AD patients, ROS levels are elevated, ROS can further stimulate the production of inflammatory factors, exacerbate the deposition of Aβ, and neuronal damage, thus aggravating the pathologic process of AD. Altered BBB permeability is an important driver in aggravating neurological diseases, and ROS can directly affect BBB permeability. On the one hand, ROS cause a decrease in the expression of tight junction proteins (ZO-1, occludin) in endothelial cells, which affects BBB permeability; on the other hand, ROS can directly oxidize lipids and proteins in cell membranes, leading to lipid peroxidation and alteration of protein structure, which increases the permeability of the BBB (Yang et al., 2021; Kim Y. et al., 2022). In addition, ROS were able to activate the NF-κB pathway and release inflammatory factors such as tumor necrosis factor α (TNF-α) and interleukin 1β (IL-1β), which further disrupted the integrity of the BBB (Gu et al., 2021). Oxidative stress has a complex relationship with CI/RI, VaD and AD, and will also interact with other mechanisms to influence neurological disorders. Here, we collate previous studies to summarize the potential role of oxidative stress in the development of three neurological disorders, namely CI/RI, VaD and AD, so as to provide a theoretical basis for further subsequent studies (Table 1).

#### 3.2.1 Oxidative stress and CI/RI

Reduced oxygen supply to brain tissue after ischemia, altered mitochondrial membrane potential, and affected oxidative phosphorylation result in reduced ATP production, leading to the production of reactive oxygen species such as superoxide anion (O^2−^), hydroxyl radical (·OH), reactive nitrogen species (RNS), and nitric oxide (NO), which promotes inflammation, damages proteins, cell membranes, and the DAN, and causes damage to the organism (Kong et al., 2020; Trujillo-Rangel et al., 2022). Pnn is a serine- and arginine-rich protein that plays multiple roles in regulating cell differentiation, proliferation, and migration (Joo et al., 2014). Studies have shown that Pnn deficiency exacerbates oxidative stress in neurons and exacerbates brain CI/RI in mice (Hsu et al., 2022). Nitric oxide synthase (NOS) is responsible for the production of nitric oxide (NO). NOS can be classified into three main categories: endothelial NOS (eNOS), neuronal NOS (nNOS) and inducible NOS (iNOS) (Pradhan et al., 2018). NOX located in the cell membrane is another source of ROS during the reperfusion phase. The NOX enzyme uses oxygen as the final electron acceptor via NADPH and immediately produces O^2−^. O^2−^ crosses the membrane via the anion channel pore, resulting in the degradation of NO, the formation of peroxynitrite, and the nitration of protein tyrosine (Wu et al., 2018). Melatonin attenuates cerebral CI/RI damage in mice by upregulating SIRT3 expression and activating SIRT3 signaling after middle cerebral artery occlusion (Liu et al., 2019). Diosgenin increases protein expression of SIRT2, T-Nrf2, N-Nrf1, NQO1 and HO-527 *in vivo* and *in vitro*, and activates the SIRT2/Nrf2 signaling pathway to inhibit oxidative stress to attenuate brain CI/RI injury (Lee et al., 2012). Sanggenon C (SC), a flavonoid present in Cortex Mori, inhibits RhoA-ROCK signaling, reduces inflammation and oxidative stress levels in middle cerebral artery occlusion (MCAO) reperfused rats, and reduces post-CI/RI is injury (Zhao and Xu, 2020). Parthenolide (PN) is a sesquiterpene lactone extracted from *Tanacetum parthenium*, which can alleviate OGD/R-induced oxidative stress and neuroinflammatory injury through activation of the Akt/GSK-3β signaling pathway, and exert a protective effect on PC12 cells. Puerarin (PUE) is one of the main components of the traditional Chinese medicine Puerariae Lobatae Radix. A recent study showed that PUE activated the PI3K/Akt/Nrf2 signaling pathway, induced dissociation of the Nrf2-Keap1 complex and accelerated nuclear translocation of Nrf2, thereby intervening in oxidative stress and protecting neuronal cells in both the middle cerebral artery occlusion/reperfusion (MCAO/R) model rat and the oxygen–glucose deprivation/reperfusion (OGD/R) cell model (Zhang et al., 2023). Sodium butyrate (NaB) Significantly increased the protein expression levels of Jak2, STAT3 and SOD activity, and decreased MDA activity and IL-1β, TNF-α, IL-8 levels in CI/RI mice. Meanwhile, the positive neurons of TUNEL staining were significantly reduced, i.e., NaB prevented oxidative stress, inflammatory response and neuronal apoptosis by inhibiting the JNK/STAT pathway and reduced neuronal injury after CI/RI (Wang et al., 2019).

Micro RNA (miRNA) is a non-coding small RNA molecule with about 18–25 nucleotides, which has the role of regulating gene expression, participating in cell growth and differentiation, regulating stress and immunity, etc. miRNAs have been associated with many diseases, such as cancer, cardiovascular diseases, and neurodegenerative diseases, etc. In the central nervous system (CNS), 15 miRNAs were found to be up-regulated and 44 miRNAs were found to be up-regulated in the presence of CI/RI. In the central nervous system, miRNAs are involved in synapses, myelin sheaths and cerebral angiogenesis. Fifteen miRNAs were found to be up-regulated and 44 miRNAs were found to be down-regulated under CI/RI conditions (Min et al., 2015). It was shown that miR-489-3p was up-regulated and SIRT1 was down-regulated in the brain tissues of rats with transient middle cerebral artery occlusion (tMCAO) model, whereas down-regulation of miR-489-3p or up-regulation of SIRT1 ameliorated behavioral dysfunctions, lowered the level of oxidative stress, and reduced neuronal damage and apoptosis in rats (Song L. et al., 2022). lncRNA 22 prime to Xist (FTX) inhibits apoptosis and oxidative stress through the miR-186-5p/MDM4 pathway, thereby attenuating CI/RI (Xiao et al., 2022). In the rat CI/RI model and SH-SY5Y cells using the hypoxia/reoxygenation (H/R) model, NOX2 expression was significantly increased, whereas the expression level of miR-652, a potential target of NOX2, was significantly decreased in brain tissue and plasma (Zuo et al., 2020). In contrast, miR-652 significantly reduced NOX2 expression and ROS generation, as well as tissue damage in brain tissues of CI/RI rats (Zuo et al., 2020). Lignans, a major component of Syringa pinnatifolia Hemsl, activated the Nrf2/HO-1 signaling pathway, increased SOD and GPx activities, decreased MDA levels, and inhibited the expression of NOX2 and NOX4, which significantly ameliorated the neurological function of brain tissues and pathological injuries, and it alleviated the CI/RI and OS, and reduced the volume of cerebral infarction (Hao et al., 2023). Modeling using the middle cerebral artery occlusion (MCAO) method showed a significant increase in the levels of phosphorylated JNK, p38, and ERK in rats of the model group, and intervention with fucoidan resulted in a decrease in the levels of phosphorylated JNK, p38, and ERK, as well as an increase in the activity of SOD, a decrease in the levels of MDA, and a decrease in the damage caused by CI/RI in the rats, which might be related to the fact that Fucoidan inhibits the MAPK pathway (Che et al., 2017).

#### 3.2.2 Oxidative stress and VaD

The development of VaD is inextricably linked to oxidative stress. The excessive accumulation of ROS oxidises unsaturated fatty acids on neuronal cell membranes, thereby causing cellular damage, triggering an inflammatory response, and resulting in vascular damage and dysfunction. This ultimately leads to a significant loss of neurons and inadequate blood supply to the brain, which in turn gives rise to VaD (Li Z. et al., 2011). Oxidative stress induced by the overactivation of NADPH oxidase plays an important role in the pathology of VaD. Studies have shown that the expression of NADPH oxidase is increased in the brain tissues of VaD rats, and cognitive deficits in VaD rats can be attenuated by inhibiting the activity of NADPH oxidase, suggesting that NADPH oxidase plays an important role in the pathogenesis of VaD (Choi et al., 2014). It was found that Puerarin could significantly reduce MDA levels and increase GSH-Px levels in the hippocampus and frontal cortex of VaD model rats, and improve the ability of rats to learn to improve memory. The study also used Puerarin to treat H_2_O_2_-stimulated SH-SY5Y cells, and the results showed that Puerarin could up-regulate key genes related to the expression of antioxidant proteins, such as Nrf2, FOXO1, FOXO3, and FOXO4, and alleviate oxidative stress damage (Zhang et al., 2015). It was found that resveratrol shortened the escape latency and distance of VaD model rats in the Morris water maze experiment, and extended the percentage of time spent in the target quadrant and the percentage of distance swum during the orientation voyage, suggesting that resveratrol improves the learning and memory abilities of VaD rats (Ma et al., 2013). Further experiments revealed that resveratrol decreased MOD levels in the hippocampus and cerebral cortex and increased SOD and GSH levels in VaD model rats (Ma et al., 2013). It was found that *Artemisia annua* Linn’e could reduce the level of oxidative stress and neuroinflammation, protect neurons, and attenuate cortical microvascular and BBB injury after chronic cerebral hypoperfusion (CCH) by activating the Nrf2/Keap1/ARE signaling pathway, which could protect the Neurovascular unit (NVU) integrity, thereby improving cognitive function in VaD model animals with bilateral common carotid artery occlusion (BCCAO) (Kim et al., 2023). Ligusticum chuanxiong lactone (LIG) is one of the main active ingredients of Angelica sinensis, and the results of Morris water maze showed that LIG effectively improved the learning and memory deficits in VaD rats, which may be related to the activation of the AMPK/SIRT signaling pathway by LIG to reduce oxidative stress damage and anti-apoptosis (Peng et al., 2022). Amorphous selenium nanodots (A SeNDs) inhibited oxidative stress damage in vascular dementia rat neurons by activating the NMDAR pathway (Zhu et al., 2023). Glycyrrhizic acid (GA) exerted antioxidant effects by restoring mitochondrial complexes I and IV, enzymatic and non-enzymatic antioxidant activities, and reducing ROS production through modulation of the GSK3β/Nrf2 signaling pathway, and exerted a protective effect against chronic cerebral hypoperfusion in VaD rats (Sathyamoorthy et al., 2020). Another study showed that GA improved learning memory ability, attenuated cellular damage, decreased MDA levels, and increased SOD activity in VaD model rats, which may be related to the inhibition of oxidative stress and the reduction of the current intensity of voltage-gated sodium channels (VGSC) in pyramidal neurons of the hippocampal CA1 region (Guo et al., 2016).

#### 3.2.3 Oxidative stress and AD

The pathogenesis of AD is a complex process, with a number of well-known mechanisms involved, including Aβ deposition, tau protein hyperphosphorylation, oxidative stress, neuroinflammation, cholinergic damage and metal ion disorders. The formation of senile plaques by Aβ deposition and the formation of neurofibrillary tangles by tau protein hyperphosphorylation represent the two most significant pathological manifestations of AD. Furthermore, the interaction between Aβ and ROS serves to exacerbate the pathological process of AD (Tamagno et al., 2021). Aβ is a polypeptide cleaved by Amyloid precursor protein (APP). APP is cleaved by α-secretase (ADAM10) or β-secretase (BACE1) to form an intracellular structural domain, which is further cleaved by γ-secretase. APP is cleaved by α-secretase and γ-secretase to produce P3 fragments. This pathway is designated the non-amyloid pathway, whereas the amyloid pathway entails the proteolytic processing of APP by β-secretase and γ-secretase, ultimately resulting in the generation of the Aβ fragment (Tarasoff-Conway et al., 2015; Koelsch, 2017). A competitive relationship exists between α-secretase and β-secretase. Studies have demonstrated that β-secretase levels and activity are markedly elevated in the brains of patients diagnosed with AD (Koelsch, 2017). ROS promote Aβ production by affecting the activity of β-secretase and γ-secretase (amyloid pathway). In turn, Aβ can bind directly to mitochondria, impairing mitochondrial function, affecting the electron transport chain, and increasing ROS production (Mehrabadi and Sadr, 2020). Aβ deposition can also lead to peroxidation of membrane lipids, which not only damages the integrity of cell membranes, but also further promotes the production of ROS. At the same time, Aβ deposition affects the expression and activity of antioxidants such as SOD and glutathione peroxidase, reducing the body’s antioxidant capacity (Athari Nik Azm et al., 2018). In conclusion, ROS and Aβ interact with each other, thereby exacerbating AD pathology. Magnoflorine, a quaternary salt porphyrin alkaloid, was found to reduce Aβ-induced apoptosis and decrease ROS levels through the JNK signaling pathway and improve learning memory in AD mice (Zhong et al., 2023). Ginsenoside Rk3 inhibited apoptosis and glial cell activation by activating the AMPK/Nrf2 signaling pathway, increasing the levels of glutathione reductase (GSH) and superoxide dismutase (SOD), and decreasing the levels of malondialdehyde (MDA), thereby improving the learning and memory abilities of APP/PS1 mice (She et al., 2023). BisdemethoxyCurcumin (BDMC) is a classical derivative of curcumin with neuroprotective effects. After intervention with BDMC, APP/PS1 mice showed improved cognitive function, decreased Aβ deposition, reduced oxidative stress levels, increased neuron numbers, and elevated SIRT1 expression levels. However, after intervention with an inhibitor of SIRT1, it was found that the above results were inextricably linked to the upregulation of SIRT1, i.e., BDMC could alleviate cognitive deficits in APP/PS1 mice by upregulating SIRT1 to achieve an anti-oxidative stress effect (Xu et al., 2020). Sesamin and sesamol reduced the level of oxidative stress in H_2_O_2_-induced human neuroblastoma (SH-SY5Y) through the SIRT1-SIRT3-FOXO3a signaling pathway. Imperatorin, a major constituent of Prehistoria, has the ability to promote Nrf2 nuclear translocation and increase the expression levels of Nrf2, NQO-1, and HO-1, thereby reducing oxidative stress, lowering mitochondrial membrane potential, and ameliorating cobalt chloride-induced apoptosis (Liao et al., 2023).

## 4 Progress in clinical research

As previously stated, the effects of mitophagy and oxidative stress on the pathological conditions of CI/RI, VaD and AD have been extensively investigated through a multitude of cellular and animal experimental studies. Nevertheless, it is important to note that animal and cellular models cannot fully replicate the complex and multifaceted characteristics of real disease states. Therefore, we summarize the studies of the last decade in the clinic and summarize the safety and resistance of the first-line drugs that are under development or have been approved. Currently, tPA and endovascular thrombectomy are effective treatments for stroke (Powers et al., 2019). However, there is a strict window of time for tPA and thrombectomy, which leads to a very high rate of disability in patients with CI/RI. Unfortunately, there is still no specific drug for the treatment of VaD and AD. Although, FDA-approved clinical first-line drugs for AD include memantine, donepezil hydrochloride, and galantamine, they are unable to fundamentally block the pathological process of AD (Table 2).

**Table 2:** Summary of clinical studies in recent years.

Drug	Mechanism of Action	Therapeutic Purpose	NCT Number	Status	Country	Phases	Start Year	Estimated year of completion
Butylphthalide soft capsule	DL-3-n-butylphthalide Improves the oxidative stress response of the nervous system Inhibits neuronal apoptosis and autophagy Regulates the	Cognitive function enhancer	NCT03804229	Active, not recruiting	China	Phase 3	2019	2024
Akatinol Memantine	Reduce oxidative stress, β-amyloid plaque formation, increase brain-derived neurotrophic factor (BDNF), inhibit glutamate release, promote cholinergic neuron regeneration and repair	The study evaluates the clinical efficacy and safety of Akatinol Memantine 20 mg (single-doses) vs. Akatinol Memantine 10 mg (double-doses) in patients suffering from moderate and moderately severe vascular dementia.	NCT03986424	Completed	Russian Federation	Phase 3	2019	2022
Tian Ma Bian Chun Zhi Gan Tablets			NCT05371639	Recruiting	China	Phase 2	2022	2025
CognivAiD	Antioxidant, anti-inflammatory	Assessing the safe use and effectiveness of CognivAiD	NCT06228638	Recruiting	Singapore	-	2023	2025
Dapagliflozin	Antioxidant, anti-inflammatory	To assess the effect of daragliflozin on cardiovascular risk in patients with ischemic cerebrovascular disease		Recruiting	Mexico	Phase 2, phase 3	2020	2025
CY6463	Anti-inflammatory	To test the safety, tolerability and pharmacokinetics of CY6463 compared to placebo in individuals aged 60 years or older with AD and common cardiovascular risk factors	NCT04798989	Terminated	United States	Phase 2	2021	2022
N-831 (Traneurocin)	–	This study was designed to evaluate the efficacy and safety of NA-83 in 126 subjects with mild cognitive impairment due to Alzheimer’s disease	NCT03538522	Completed	New Zealand	-	2018	2019
Hyperbaric oxygen therapy (HBOT)	Anti-inflammatory, reduces tua protein phosphorylation, reduces Aβ deposition	Exploring the Potential Beneficial Effects of HBOT on Precursor AD	NCT05349318	Recruiting	Israel	Not Applicable	2022	2025
Donepezil hydrochloride (Aricept)	Regulates mitophagy, anti-inflammatory, antioxidant, reduces Aβ deposition	To assess the efficacy and safety of Aricept in patients with VaD	NCT00165763	Completed	Philippines	Phase 4	2005	2009
Metformin	Reduction of tau hyperphosphorylation and Aβ deposition	To investigate the effect of insulin sensitizer metformin on AD biomarkers (tau protein, Aβ)	NCT01965756	Completed	United States	Phase 2	2013	2017
Donepezil	Regulates mitophagy, anti-inflammatory, antioxidant, reduces Aβ deposition	To investigate whether Allison can enhance the ability to remember functional task steps and the actual ability to perform tasks related to real-life independence	NCT00457769	Unknown status	United States	-	2007	2021

Desmoteplase is a fibrin-dependent fibrinogen activator with a favourable safety profile in clinical practice. Nevertheless, clinical studies have revealed no improvement in patients presenting with an ischaemic stroke and cerebral artery occlusion, with episodes lasting in excess of 3 h (Albers et al., 2015). Argatroban is a selective thrombin inhibitor that is widely used in the treatment of acute ischemic stroke in Asian countries such as China and Japan. Studies have reported that Argatroban plus alteplase, a recombinant tissue-type fibrinogen activator, is beneficial for patients with acute ischemic stroke (AIS) (Jang et al., 1990; Morris et al., 2001). Argatroban treatment in combination with intravenous alteplase was not significantly different in all scores at 90 days compared to alteplase alone (Chen et al., 2023). Edaravone is a free radical scavenger with antioxidant, neuroprotective and anti-inflammatory effects (Sriram et al., 2016; Dang et al., 2022). Administration of edaravone within 48 h of endovascular revascularization improves functional independence at discharge, reduces in-hospital mortality, and decreases post-admission intracranial hemorrhage, according to a study that included 10,000 patients with acute ischemic stroke (Enomoto et al., 2019). In recent years, cell therapy has achieved better therapeutic effects in animal models of acute ischemic stroke, and pluripotent adult progenitor cells are a kind of bone marrow-derived allogeneic cell therapeutic product with the role of the immune system. Studies have shown that pluripotent adult progenitor cells are safe, well tolerated and have few adverse effects in the treatment of acute ischemic stroke (Hess et al., 2017). Although there was no significant difference between the pluripotent adult progenitor cell treatment group and the control group at 90 days after acute ischemic stroke, clinical trials in the early time window of stroke (<36 h) are still anticipated (Hess et al., 2017). Results of a prospective cohort study showed that MDA levels were significantly higher in patients with severe stroke compared with those with moderate and mild acute ischemic stroke (Elhamrawy et al., 2023). N-acetylcysteine (NAC) can react with ROS, and oral administration of NAC in the early stage of acute ischemic stroke significantly reduced the serum levels of interleukin 6 (IL-6), soluble intercellular adhesion molecule-1 (sICAM-1), nitric oxide (NO), malondialdehyde (MDA), and increased the levels of antioxidant biomarkers, such as SOD, GPx and total thiol group (TTG). The levels of antioxidant biomarkers such as SOD, GPx and total thiol group (TTG) were significantly increased to improve the damage caused by ischemic stroke in patients (Sabetghadam et al., 2020). Minocycline is a semi-synthetic tetracycline, which has been found in recent years to exert neuroprotective effects by mitigating oxidative stress damage (Naderi et al., 2017; Zhao et al., 2023). The results of several clinical studies confirm the role of minocycline in ameliorating ischemic stroke injury without death, myocardial infarction, recurrent stroke, or hemorrhagic transformation during follow-up (Lampl et al., 2007; Padma Srivastava et al., 2012; Amiri-Nikpour et al., 2015; Ortiz et al., 2020).

To date, no specific drug has been developed for the treatment of VaD, and approaches to preventing and treating VaD have focused on controlling vascular risk factors. Clinical trials have shown that hypertension is a risk factor for VaD and that early treatment of hypertension can reduce the risk of VaD and slow the disease process (Tariq and Barber, 2018). However, statins for hyperlipidemia or acetylsalicylates for antiplatelet agents did not have a significant effect on disease progression in the relief of VaD (Appleton et al., 2017; Lazashvili et al., 2021). Pimovanserin is a 5-hydroxytryptamine receptor modulator that acts primarily as a selective 5-hydroxytryptamine receptor subtype 2A (5-HT2 amp) inverse agonist and antagonist. In a study of pimovanserin in the treatment of dementia-related psychiatric disorders, 105 patients were treated with pimovanserin and 112 patients were treated with placebo. Twelve (13%) of 95 patients in the pimovanserin group and 28 (28%) of 99 patients in the placebo group relapsed. Adverse events occurred in 43 (41.0%) in the pimovanserin group and 41 (36.6%) in the placebo group. The results of the trial suggest that the risk of relapse in patients with dementia-related psychosis who responded to pimovanserin is lower with continued medication than with discontinuation (Tariot et al., 2021). Butylphthalide has the effect of vasodilatation, regulating blood circulation, increasing the blood flow of cerebral blood supply arteries, and regulating mitochondrial functions (Niu et al., 2021; Gao et al., 2022). Therefore, it is often used in clinical practice to treat patients with VaD. Idebenone can eliminate oxygen free radicals, resist oxidation, enhance brain mitochondrial function, and improve brain function and metabolic state (Pradhan et al., 2021; Lee et al., 2022), and is also an important drug in the clinical treatment of cerebrovascular diseases. Clinical studies have shown that butylphthalide combined with ibenzoquinone can effectively reduce serum inflammatory factor levels, regulate vascular endothelial function, alleviate the degree of dementia, and improve cognitive function and daily activities in patients with VaD (Zhang et al., 2024). Tianzhi granule (TZ), an approved Chinese herbal medicine by China FDA for VaD, whose main ingredients include geniposide, gastrodin, baicalinand, rutin so on. A total of 543 mild-to-moderate VaD A total of 543 patients with mild-to-moderate VaD were enrolled, of whom 242 were taking TZ granules, 241 were taking donepezil, and 60 were taking placebo. Both TZ and donepezil showed small but significant improvements, with significant differences between the TZ group and the placebo group, and no significant differences were observed between TZ and donepezil (Shi et al., 2020). Another study found that Shenma YIzhi formula (SYF) significantly improved MMSE, National Institutes of Health Stroke Scale (NIHSS) and CM-SS scores in patients with VaD (Zhang et al., 2020). Mechanisms that may be related to the improvement of vascular endothelial function, mitochondrial function and cholinergic dysfunction (Wu et al., 2019; Sun et al., 2021). Qi Fu Yin consists of *Panax ginseng*, Angelica sinensis, Rehmannia glutinosa, Glycyrrhiza uralensis, Polygala tenuifolia, Atractylodes macrocephala and Semen ziziphi spinosae composition. Qi Fu Yin improved the scores of cognitive function assessment scales such as Hasegawa Dementia Scale (HDS) and the Brief Mental State Examination (MMSE) in patients with AD and VaD (Wang et al., 2021), which might be related to the fact that Qi Fu Yin promoted the anti-inflammatory factors IL-5, IL-10 and G-CSF, decreased the levels of the pro-inflammatory factor IFN-γ, reduced Aβ deposition, and regulated the diversity and abundance of intestinal flora (Xiao et al., 2022; He et al., 2023; Yang et al., 2023a). Boyang-Hwano-Tang (BHT) is a Chinese herb widely used in Korea and China for the treatment of ischemic stroke. Meta-analysis results showed that BHT has a clinical role in improving cognitive function in patients with vascular dementia (Kim D. W. et al., 2022). Naoqingzhiming is the first novel Class I natural drug approved in China with potential for the treatment of VaD, and its active ingredient is echinacoside. Single ascending doses of Naoqingzhiming tablets (180–2,160 mg) were well tolerated in all enrolled subjects, with no serious adverse events and no adverse events leading to withdrawal from the study (Lin et al., 2022). Hyperbaric oxygen (HBO) therapy has been used to treat a variety of diseases; however, HBO is not recommended as a treatment for VaD in existing guidelines. Studies have found that HBO therapy improves cognitive function in patients with VaD, and the mechanism may be related to elevated serum human protein levels (Xu et al., 2019).

Memantine, a glutamate NMDA receptor antagonist, is approved by the U.S. FDA for the treatment of moderate and severe AD. Meat analyses have shown that memantine has a small interventional effect in patients with moderate-to-severe AD, but no effect in patients with mild AD (Azhar et al., 2022). Aducanumab and Lecanemab are both humanized monoclonal antibodies. Aducanumab has a high affinity for both soluble and insoluble Aβ, while Lecanemab has a high affinity for soluble Aβ. A recent clinical study enrolled 1,795 AD patients, of whom 898 received lecanemab and 897 received placebo. In patients with early-stage AD, lecanemab reduced Aβ levels and showed a smaller decline in cognitive and functional clinical measures compared to placebo at 18 months (van Dyck et al., 2023). Aducanumab reduced AD pathologic markers with dose- and time-dependent reductions in a study involving 348 sites in 20 countries (Budd Haeberlein et al., 2022). Suvorexant is a dual orexin receptor antagonist that has been approved by the FDA for the treatment of insomnia. Studies have shown that suvorexant has the ability to reduce the concentration of Aβ and phosphorylated tau in the central nervous system (Lucey et al., 2023). Therefore, Suvorexant is expected to be a future drug for the treatment of AD. Masitinib is an oral tyrosine kinase inhibitor that targets activated cells of the neuroimmune system (mast cells and microglia). Masitinib (4.5 mg/kg/day) may be beneficial in patients with mild to moderate AD, whereas efficacy results in an independent parallel group of patients treated with Masitinib (4.5 mg/kg/day) have not been conclusive. Efficacy results are inconclusive and subsequent experimental studies have been conducted (Dubois et al., 2023).

## 5 Summary and prospect

The interaction between oxidative stress and mitophagy is critical for the maintenance of intracellular stability and organismal health, and is important for understanding the mechanisms of a wide range of diseases as well as for the development of new therapeutic approaches. In this review, we summarize many drugs that are beneficial for CI/RI, VaD and AD. The targeting of multiple pathophysiological factors may prove to be an effective approach in the treatment of neurological disorders. Natural medicines have been demonstrated to alleviate pathological impairments and cognitive deficits in CI/RI, VaD, and AD via a multitude of mechanisms. Nevertheless, the majority of these pharmaceuticals are still in the preliminary research phase. Consequently, it is essential to exercise caution in awaiting the findings of forthcoming clinical trials.

Hypertension represents a significant risk factor for the development of CI/RI, VaD, and AD. The chronic elevation of blood pressure results in an increase in the wall-to-lumen ratio, which in turn leads to a reduction in lumen diameter, a state of cerebral hypoperfusion, and the development of vascular remodeling. After the onset of CI/RI, there is an increase in the levels of inflammatory factors and ROS, which cause further vascular and neurological damage that may evolve into VaD. Inadequate cerebral perfusion exists in both VaD and AD. Prolonged cerebral underperfusion leads to Aβ deposition and abnormal phosphorylation of tau proteins, which may be due to the inflammatory response and oxidative stress induced by ischemia and reperfusion exacerbating the AD pathological process (Pluta et al., 2021). Experiments have demonstrated that ischemia exacerbated cognitive deficits, increased Aβ deposition, tau protein phosphorylation, and aggravated neuronal damage in the hippocampus of AD rats (Li J. et al., 2011; Ułamek-Kozioł et al., 2016). A strict time limit is in place for the use of tPA, a potent drug for CI/RI. As yet, no potent drug has been identified for VaD and AD. A significant number of clinical trials, many of which yielded unsatisfactory results, have been terminated without success. It is crucial to identify early indicators of CI/RI, as well as molecular markers of VaD and AD, and integrate them into clinical diagnosis. This approach enables the implementation of prompt interventions, thereby halting the progression of these conditions and alleviating the burden of subsequent treatments (Ali et al., 2023). Many drug molecules are unable to act on the brain due to the presence of the BBB. In recent years, there has been uninterrupted research on novel nanomaterials, and a novel lipoprotein-like nanocomposite (RLA-rHDL@ANG) consisting of recombinant high-density lipoprotein (rHDL) and APOE-derived peptide (RLA) has therapeutic potential for the treatment of AD. It has been shown that RLA-rHDL@ANG efficiently passes through the BBB and accumulates in the brain. In addition, it has a high binding affinity for both Aβ monomers and oligomers, thus preventing Aβ aggregation, and promotes efficient Aβ degradation by microglia and neurons through lysosomal transport and elimination methods (Wang et al., 2023), which also gives us new inspiration to build on existing research to find materials targeting reduction of oxidative stress and enhancement of mitophagy, which may be a common vision for the treatment of CI/RI, VaD, and AD. The gut is the second brain of human beings, and many studies have demonstrated the close relationship between intestinal flora and neurological disorders. *Prevotella histicola* upregulates the expression of synapse-associated proteins and neurotrophic factors, and downregulates the levels of pro-inflammatory factors to attenuate nerve damage in VaD rats (Duan et al., 2023); *Lactobacillus* and *Bifidobacterium* improves learning memory in Aβ_1-42_-induced AD rats, which may be related to the reduction of oxidative stress levels in the rat brain (Athari Nik Azm et al., 2018). This suggests that flora transplantation or taking probiotics may be used as a therapeutic approach to treat neurological diseases. However, the current research on intestinal flora is not deep enough, and there are still a lot of problems, such as the number of flora is complicated, there are differences in the population, and the mechanism of the flora metabolites and diseases is still unclear, etc. We still need to carry out more in-depth research to address the current problems. In addition, small vessel disease (SVD) or small vessel dysfunction has become a hot research topic in recent years. Cerebral small vessel disease (SVD) is considered to be the most important vascular factor contributing to cognitive decline and dementia, and the results of a 14-year follow-up study suggest that SVD precedes the progression of dementia (including VaD, AD, and mixed AD/VaD) and may contribute to dementia development (Jacob et al., 2023). SVD or small vessel dysfunction is closely related to metabolic diseases, therefore, in combination with metabolic diseases, starting from improving small vessel function may be a means to treat neurological diseases. There are a wide variety of miRNAs expressed in the brain, and different miRNAs play key roles in neural development, synapse formation, and neural signaling. For example, miR-134 plays an important role in regulating synaptic development and plasticity, especially in learning and memory (Wayman et al., 2008). miR-132 is associated with neuroplasticity and cognitive function, especially in memory formation and regulation (Numakawa et al., 2011). Altered miR-29 expression in AD is associated with Aβ metabolism (Hébert et al., 2008; Lei et al., 2015). The functions of these miRNAs in the nervous system are complex and diverse, and they are involved in physiological and pathological changes in the brain by precisely regulating gene expression. Therefore, we may still discover more miRNAs and their specific roles in the brain as our research progresses. In conclusion, we still need a deeper understanding of the molecular mechanisms between OS and mitophagy, as well as the roles played by these two mechanisms in different disease states, to provide new strategies for the treatment of neurological diseases.

## Author contributions

YL: Conceptualization, Investigation, Methodology, Visualization, Writing – original draft, Writing – review & editing. ZM: Investigation, Methodology, Visualization, Writing – original draft, Writing – review & editing. YH: Methodology, Writing – review & editing. BJ: Conceptualization, Methodology, Writing – review & editing. JY: Methodology, Writing – review & editing. YC: Methodology, Writing – review & editing. JZ: Methodology, Writing – review & editing. ML: Methodology, Writing – review & editing. HW: Funding acquisition, Methodology, Resources, Supervision, Writing – original draft, Writing – review & editing.
