# Corrigendum: Mechanisms of mitophagy and oxidative stress in cerebral ischemia–reperfusion, vascular dementia, and Alzheimer's disease

**DOI:** 10.3389/fnmol.2024.1507345

**Published:** 2024-12-04

**Authors:** Yujie Lyu, Zhipeng Meng, Yunyun Hu, Bing Jiang, Jiao Yang, Yiqin Chen, Jun Zhou, Mingcheng Li, Huping Wang

**Affiliations:** ^1^Gansu University of Chinese Medicine, Lanzhou, China; ^2^Key Laboratory of Traditional Chinese Herbs and Prescription Innovation and Transformation of Gansu Province, Lanzhou, China; ^3^Laboratory for TCM New Products Development Engineering of Gansu Province, Lanzhou, China; ^4^Xichang Hospital of Traditional Chinese Medicine, Xichang, China; ^5^Qujing 69 Hospital, China RongTong Medical Healthcare Group Co. Ltd, Qujing, China

**Keywords:** mitophagy, oxidative stress, cerebral ischemia/reperfusion, vascular dementia, Alzheimer's disease

In the published article, there was an error in affiliation(s) 2,3,4,5. Instead of: Yujie Lyu^1†^, Zhipeng Meng^1†^, Yunyun Hu^1^, Bing Jiang^1^, Jiao Yang^1^, Yiqin Chen^1^, Jun Zhou^2^, Mingcheng Li^3^ and Huping Wang^1,4,5*^

^1^Gansu University of Chinese Medicine, Lanzhou, China, ^2^Xichang Hospital of Traditional Chinese Medicine, Xichang, China, ^3^Qujing 69 Hospital, China RongTong Medical Healthcare Group Co. Ltd, Qujing, China, ^4^Key Laboratory of Traditional Chinese Herbs and Prescription Innovation and Transformation of Gansu Province, Lanzhou, China, ^5^Laboratory for TCM New Products Development Engineering of Gansu Province, Lanzhou, China,

It should be: Yujie Lyu^1,2,3†^, Zhipeng Meng^1†^, Yunyun Hu^1^, Bing Jiang^1^, Jiao Yang^1^, Yiqin Chen^1^, Jun Zhou^4^, Mingcheng Li^5^ and Huping Wang^1,2,3*^

^1^Gansu University of Chinese Medicine, Lanzhou, China, ^2^Key Laboratory of Traditional Chinese Herbs and Prescription Innovation and Transformation of Gansu Province, Lanzhou, China, ^3^Laboratory for TCM New Products Development Engineering of Gansu Province, Lanzhou, China,^4^Xichang Hospital of Traditional Chinese Medicine, Xichang, China, ^5^Qujing 69 Hospital, China RongTong Medical Healthcare Group Co. Ltd, Qujing, China.

In the published article, there was an error in the Funding statement. Instead of: “This study was supported by two grants from the National Natural Science Foundation of China, Project Nos. 82160862, 81960828; the Natural Science Foundation of Gansu Province, Project No. 22JR11RA113; the Science and Technology Programme of Gansu Province (Joint Research Fund) Project No. 23JRRA1526; the Fifth Batch of National Training Programme for Outstanding Clinical Talents in Traditional Chinese Medicine [Letter of Human Education in Chinese Medicine (2022) 39]; the first batch of Longyuan Youth Talent Project [CPC Gansu Provincial Committee Talent Work Leading Group (2022) 5].”

The correct Funding statement appears below.

This study was supported by two grants from the National Natural Science Foundation of China, Project Nos. 82160862, 81960828; the Natural Science Foundation of Gansu Province, Project No. 22JR11RA113; the Science and Technology Programme of Gansu Province (Joint Research Fund) Project No. 23JRRA1526; the Fifth Batch of National Training Programme for Outstanding Clinical Talents in Traditional Chinese Medicine [Letter of Human Education in Chinese Medicine (2022) 39]; the first batch of Longyuan Youth Talent Project [CPC Gansu Provincial Committee Talent Work Leading Group (2022) 5]; Key Laboratory of Traditional Chinese Herbs and Prescription Innovation and Transformation of Gansu Province; Laboratory for TCM New Products Development Engineering of Gansu Province.

The authors apologize for these errors and state that this does not change the scientific conclusions of the article in any way. The original article has been updated.

